# Brain Markers of Resilience to Psychosis in High-Risk Individuals: A Systematic Review and Label-Based Meta-Analysis of Multimodal MRI Studies

**DOI:** 10.3390/brainsci15030314

**Published:** 2025-03-17

**Authors:** Guusje Collin, Joshua E. Goldenberg, Xiao Chang, Zhenghan Qi, Susan Whitfield-Gabrieli, Wiepke Cahn, Jijun Wang, William S. Stone, Matcheri S. Keshavan, Martha E. Shenton

**Affiliations:** 1 Department of Psychiatry, Radboud University Medical Center, 6525 GA Nijmegen, The Netherlands; 2 Donders Institute for Brain, Cognition and Behaviour, Radboud University, 6525 EN Nijmegen, The Netherlands; 3 McGovern Institute for Brain Research, Department of Brain and Cognitive Sciences, Massachusetts Institute of Technology, Cambridge, MA 02139, USA; 4 Psychiatry Neuroimaging Laboratory, Department of Psychiatry, Brigham and Women’s Hospital, Harvard Medical School, Boston, MA 02115, USA; 5 Institute of Science and Technology for Brain-Inspired Intelligence, Fudan University, Shanghai 200433, China; 6 Key Laboratory of Computational Neuroscience and Brain-Inspired Intelligence, Fudan University, Ministry of Education, Shanghai 200433, China; 7 Department of Psychology, Northeastern University, Boston, MA 02115, USA; z.qi@northeastern.edu; 8 Department of Communication Sciences and Disorders, Northeastern University, Boston, MA 02115, USA; 9 Department of Psychiatry, Massachusetts General Hospital, Harvard Medical School, Boston, MA 02114, USA; 10 Department of Psychiatry, University Medical Center Utrecht, 3584 CG Utrecht, The Netherlands; 11 Altrecht Mental Health Institute, 3512 PG Utrecht, The Netherlands; 12 Shanghai Key Laboratory of Psychotic Disorders, Shanghai Mental Health Center, Shanghai Jiao Tong University School of Medicine, Shanghai 200030, China; 13 Department of Psychiatry, Beth Israel Deaconess Medical Center, Harvard Medical School, Boston, MA 02115, USA; 14 Department of Radiology, Brigham and Women’s Hospital, Harvard Medical School, Boston, MA 02115, USA

**Keywords:** resilience, psychosis, high risk, clinical high risk, familial high risk, neuroimaging, MRI, multimodal, brain markers

## Abstract

**Background/Objectives**: Most individuals who have a familial or clinical risk of developing psychosis remain free from psychopathology. Identifying neural markers of resilience in these at-risk individuals may help clarify underlying mechanisms and yield novel targets for early intervention. However, in contrast to studies on risk biomarkers, studies on neural markers of resilience to psychosis are scarce. The current study aimed to identify potential brain markers of resilience to psychosis. **Methods**: A systematic review of the literature yielded a total of 43 MRI studies that reported resilience-associated brain changes in individuals with an elevated risk for psychosis. Label-based meta-analysis was used to synthesize findings across MRI modalities. **Results**: Resilience-associated brain changes were significantly overreported in the default mode and language network, and among highly connected and central brain regions. **Conclusions**: These findings suggest that the DMN and language-associated areas and central brain hubs may be hotspots for resilience-associated brain changes. These neural systems are thus of key interest as targets of inquiry and, possibly, intervention in at-risk populations.

## 1. Introduction

Resilience has been defined as “the human ability to adapt in the face of tragedy, trauma, adversity, hardship, and ongoing significant life stressors” [1] (p. 227). Although various other definitions exist in the literature, the common thread among them is the ability to adapt to adversity in a such a manner that psychological and societal functioning are preserved [2,3,4,5,6,7].

Resilience is an important concept in early-psychosis research, as most individuals (nearly 90%) who have a familial high risk (FHR) for psychosis never develop a psychotic episode [8]. Similarly, the majority of individuals (70–80%) who meet criteria for a clinical high risk (CHR) for psychosis (i.e., subthreshold psychotic symptoms combined with functional decline) do not progress to full psychosis [9,10]. While research efforts in the field tend to focus on establishing psychosis risk markers, identifying markers of resilience, and e.g., incorporating them into psychosis-prediction tools [11] or combining them with AI [12], may promote early recognition [13] and thereby improve prognosis. Moreover, identifying neural systems associated with resilience to psychosis may guide efforts to develop novel interventions to target these systems for therapeutic or preventive benefit [14].

In the social sciences, there is a long history of research on resilience in the face of adversity, including familial predisposition to psychosis [5,15,16,17,18,19,20]. In fact, some of the earliest of these studies were conducted with children from parents diagnosed with schizophrenia [20]. These studies, pioneered by Garmezy in the 1970s [21], suggested that “many of these children were “stress-resistant” or “resilient” and capable of living productive lives and adapting to life stressors, despite having a heightened risk for developing a serious mental illness” [2]. More recent work developed a dynamic model of resilience in the presence of (risk for) mental illness as a multifaceted process with interacting internal and external dimensions, as well as continuously evolving life circumstances (see [2]). Internal factors in this model include stress levels, coping skills, and problem-solving abilities. In addition to (and perhaps partly underlying) these psychological and cognitive factors, there may also be neural characteristics that confer increased resilience to mental ill health. For example, studies in so-called “superagers”, who show excellent memory capacity in advanced age (which may reflect resilience to conventional pathways of aging), have identified specific regions of cortical preservation, alongside preserved cognitive performance and better overall mental health [22,23].

To explore the hypothesis that individuals who are resilient to the development of psychosis despite an at-risk profile may show specific neural characteristics that set them apart from both patients *and* healthy (i.e., non-at-risk) controls, we performed a systematic review of the literature to identify MRI studies in FHR or CHR individuals that reported markers of resilience to psychosis. Because of the sparsity of such research, we included all imaging studies regardless of the MRI modality, adapting our analytic methods to synthesize findings across modalities. To this end, we used a label-based meta-analysis, an ROI-based type of meta-analysis [24,25,26] that relies on tallying the number of times a brain region is reported in the literature as being associated with a specific finding, here being “not developing psychosis despite being at FHR or CHR to psychosis”. This approach allowed us to pool multimodal MRI findings to determine whether specific brain regions or networks are statistically overrepresented among the reported markers of resilience to psychosis across modalities. In addition, to assess putative underlying mechanisms, we incorporated methods from graph analysis to explore whether topological factors (i.e., organizational properties of the brain network) relate to the spatial distribution of resilience markers across the brain.

By identifying putative brain markers of resilience to psychosis, we aimed to provide a reference for future resilience studies. Our overall goal was to foster new hypotheses on neural factors that may confer increased resilience to psychosis and contribute to the discovery of novel targets for early intervention in at-risk individuals.

## 2. Materials and Methods

### 2.1. Systematic Review

**Figure 1:**
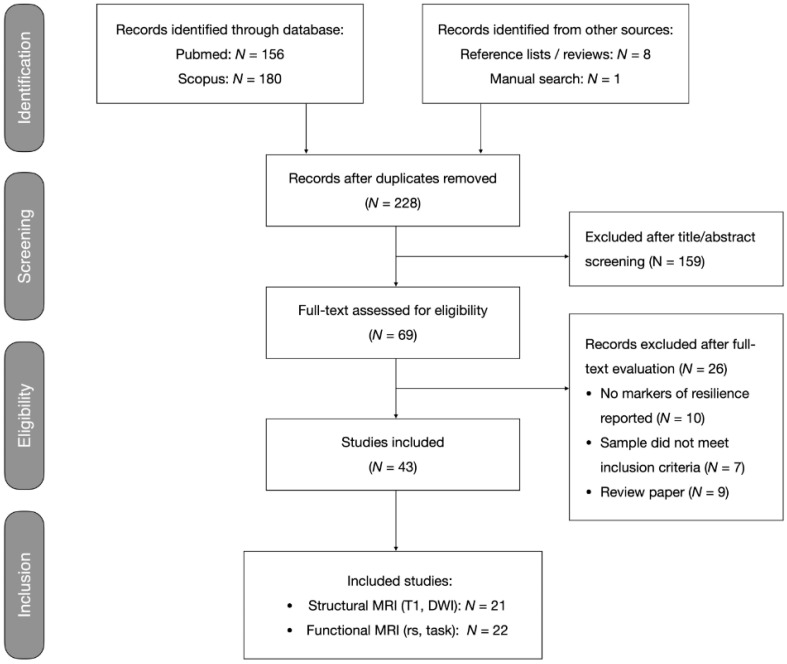
Flowchart of systematic search and selection procedures. Records excluded after full-text evaluation are listed in Supplementary Materials (Table S5).

This systematic review was performed according to guidance from the Preferred Reporting Items for Systematic Reviews and Meta-Analysis (PRISMA) [27]. This review was not pre-registered. Two investigators (G.C., J.E.G.) independently performed systematic search, selection, and critical evaluation procedures. Disagreements were discussed and resolved by consensus. Figure 1 shows the flow diagram depicting the search and selection procedures.

#### 2.1.1. Search and Selection Procedure

A comprehensive search was conducted in the PubMed and Scopus databases up to January 2020 using the following search terms: [“relatives” OR “siblings” OR “risk”] AND [“psychosis” OR “schizophrenia” OR “bipolar”] AND [“neuroimaging” OR “MRI” OR “imaging”] AND [“resilience” OR “compensatory” OR “protective”]. The retrieved records were supplemented with studies referenced by included studies or relevant review articles or as found by a manual search.

**Figure 2:**
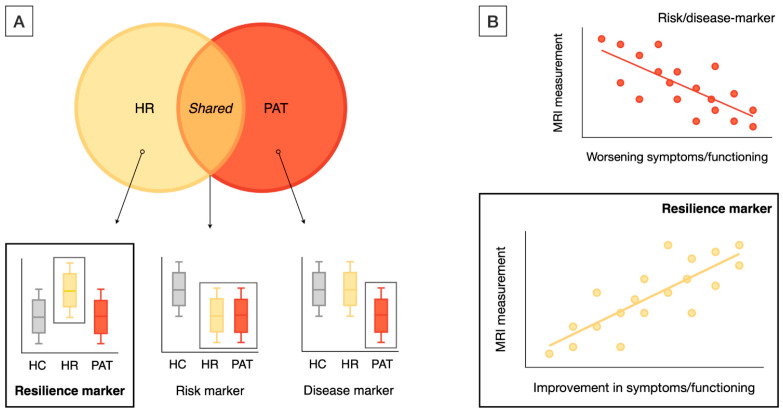
Definitions of resilience markers. Resilience markers were operationalized as MRI markers showing unique differences in HR as compared with PAT and HC (**A**) (as opposed to MRI changes shared between PAT and HR or between HC and HR) or showing correlations with positive outcomes, such as improvements in symptoms and functioning in HR cohorts (**B**). HR included both CHR and FHR individuals; HC—healthy control group; HR—high risk (i.e., either CHR or FHR) group; PAT—patient group.

Studies that met the following inclusion criteria were included: (i) original research papers written in English; (ii) neuroimaging studies that used structural MRI (sMRI), diffusion-MRI, and task-related or resting-state fMRI (task-fMRI, rs-fMRI); (iii) studies that included a high-risk (HR) group, including either CHR [28], at-risk mental state [29], or ultra high-risk [30], or FHR individuals, including first-degree relatives (FDRs) of patients with a schizophrenia spectrum or type 1 (i.e., manic-psychotic) bipolar disorder; and (iv) studies that reported neuroimaging markers of resilience to psychosis. Resilience-associated neuroimaging markers were operationalized as MRI markers showing either significant differences in the HR (i.e., either CHR or FHR) group as compared with both the patient and healthy control (HC) groups (Figure 2A) or an association with positive outcomes (e.g., improved function) in the HR samples (Figure 2B). Additional inclusion criteria for label-based meta-analysis included (i) studies that reported ROI-based findings and (ii) studies that reported results in standardized coordinate (e.g., MNI) space.

#### 2.1.2. Data Extraction

Data were extracted systematically for each publication: sample details, including the sample size, type of high-risk group, and demographics; experimental procedures, including MRI acquisition and analysis; statistical methods, including multiple comparison correction; and main findings, including regional localization based on atlas or MNI coordinates, as applicable. Only results reported as significant were considered.

#### 2.1.3. Critical Evaluation

A critical evaluation was performed on the following reliability criteria: (i) sample size over 20 participants per group (i.e., >60 participants in total), (ii) adequate motion correction [31,32,33], and (iii) appropriate multiple comparison correction. Additional information on the critical evaluation procedure is provided in the Supplementary Materials, and Table S1 provides details on the quality assessment of each included study. Given the paucity of neuroimaging findings on resilience to psychosis, studies with quality concerns were not simply excluded. Rather, their findings were weighted according to the number of putative quality issues, assigning less weight to findings from studies with quality concerns. To this end, the findings from studies with one or two concerns were weighted as 0.67 and 0.33, respectively. The quality-weighted results went into the regional and system-level tallies of resilience effects, as specified below.

### 2.2. Label-Based Meta-Analysis

#### 2.2.1. Regional Analysis

A region-wise analysis was used to assess whether the multimodal markers of resilience to psychosis converged on specific brain regions. To this end, quality-adjusted findings across imaging modalities were mapped to the Desikan–Killiany (DK) atlas (for details, see Table S4) and tallied per DK region (Supplementary Material, Table S2).

#### 2.2.2. Network-Level Analysis

To assess whether specific brain networks are associated with resilience to psychosis, DK regions were assigned to one of seven networks defined by Yeo et al. (2011), including the default mode, frontoparietal control, somatomotor, visual, limbic, ventral attention, and dorsal attention networks [34], and tallied per network. In a follow-up analysis, the Yeo et al. parcellation was adapted to include a language network, resulting in an eight-network parcellation that was separately analyzed (Supplementary Material, Figure S1).

#### 2.2.3. Graph Theoretical Analysis

To assess whether a brain region’s involvement in resilience relates to its topological role in the overall brain network, we tested regional tallies of resilience-related effects for associations with metrics of brain network organization. To compute these metrics, structural brain networks were reconstructed from an independent sample of healthy controls [35], with connections weighted according to the number of diffusion-MRI-derived tractography streamlines, and used to compute the regional strength, path length, clustering, betweenness centrality, and rich club membership. These metrics provide a measure of a region’s overall connectivity (strength), communication efficiency (path length), local cliquishness (clustering), centrality in the network (betweenness centrality), and whether they pertain to a central core of densely connected brain hubs (rich club membership) [35,36]. No standardization or normalization was applied to these metrics.

### 2.3. Statistical Analysis

#### 2.3.1. Regional and Network-Level Analyses

Permutation analysis was used to test the statistical significance of regional and network-level findings. For each of 10,000 iterations, empirical findings were randomly redistributed across regions of the DK atlas (as shown in Table S2) and tallied per region in each iteration, creating a regional null distribution of findings under the hypothesis that their localization was driven by chance. For each brain region, the sum of the empirical findings was compared with the regional null distribution and assigned a *p*-value as the proportion of random iterations that produced a sum equal to, or greater than, the empirical result. No smoothing techniques were applied. The same method was used to assess the statistical significance of the network-level results. An FDR correction was applied to all the results to correct for multiple comparisons.

#### 2.3.2. Graph Theoretical Meta-Analysis

Pearson’s correlation analysis was used to assess the associations between regional tallies of resilience-related effects and regional metrics of brain network organization. The distribution of resilience findings in the rich club versus the non-rich club regions was tested for statistical significance using a permutation analysis following the same procedures specified in Section 2.3.1.

## 3. Results

### 3.1. Systematic Review

The literature search yielded a total of 336 records, including 117 duplicates. The remaining 219 records were combined with 9 records from the manual search and cross-checking reference lists of eligible articles and review papers, which yielded a total of 228 records. The screening of the titles and abstracts yielded 69 papers for full-text evaluation. Of these, 26 publications did not meet inclusion criteria (details in Figure 1), leaving 43 papers that were selected for this review.

The 43 selected studies included 16 sMRI, 5 diffusion-MRI, 16 task-fMRI, and 6 rs-fMRI studies (the study details and main findings per imaging modality are given in Appendix A, Table A1, Table A2, Table A3 and Table A4), which comprised a total of 4732 participants, including 1455 HR individuals, 1434 patients with established psychotic illness, and 1843 HC individuals. Out of the 43 studies, 14 reported resilience-associated increases in the regional volume (*N* = 9) and/or cortical thickness (*N* = 5) or surface area (*N* = 1) (Table A1) and 5 reported increases in the structural connectivity (Table A2). No studies reported resilience-associated decreases in the cortical volume, thickness, or surface area or structural connectivity. Resilience-associated changes in the functional activity or connectivity were reported by 14 (Table A3) and 6 (Table A4) studies, respectively, and involved mainly increases in the activation or connectivity (*N* = 17). Finally, six studies reported other resilience-related effects, including changes in the regional shape (*N* = 1), structural covariance (*N* = 1) (Table A1), or global network connectivity/topology (*N* = 4) (Table A4).

### 3.2. Label-Based Meta-Analysis

#### 3.2.1. Regional Results

**Figure 3:**
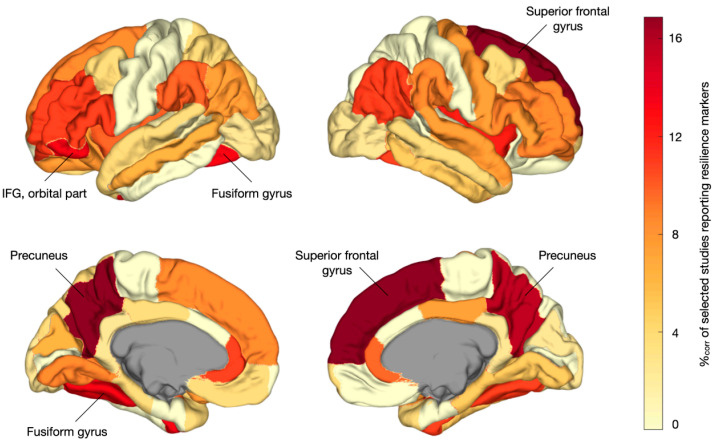
Regional localization of multimodal MRI markers of resilience.

Regionally specific results were reported by 35 studies that comprised a total of 3111 participants (i.e., 1018 in the HR, 881 in the PAT, 1212 in the HC). Figure 3 shows a pooled aggregate of the regional resilience-associated effects across the MRI modalities. In the regional permutation analysis, MRI markers of resilience were overreported among the left and right precuneus (*p* = 0.008 and *p* = 0.009, respectively); right superior frontal gyrus (*p* = 0.007), left fusiform gyrus (FG) (*p* = 0.028); and left inferior frontal gyrus (IFG), orbital part (*p* = 0.046). However, these effects did not survive an FDR correction.

The cortical plots below depict the localization of resilience markers across all the included studies. Darker colors indicate more frequent reporting in the literature as showing resilience-related effects. Regions marked by name were overrepresented among the resilience effects (*p* < 0.05, permutation testing, non-FDR significant). %_corr_—corrected percentage of studies reporting region-specific effects; IFG—inferior frontal gyrus.

#### 3.2.2. Network-Level Results

**Figure 4:**
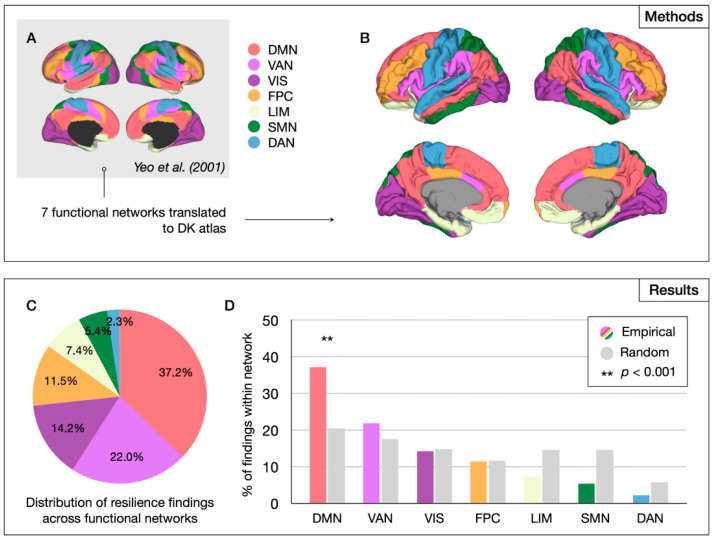
Network-level analysis of resilience markers. Methods (upper panel) and results (lower panel) of the system-level analysis. DK atlas regions were assigned to one of seven functional networks as defined by Yeo et al. (2001) [34] (**A**), resulting in a seven-network parcellation of the DK atlas (**B**). Well over a third of all resilience findings were found to be reported among the DMN regions (**C**) and the overrepresentation of the DMN among the reported resilience findings was statistically significant in permutation analysis (**D**). DMN—default mode network; FPC—frontoparietal control network; SMN—somatomotor network; VIS—visual network; LIM—limbic network; VAN—ventral attention network; DAN—dorsal attention network.

Brain regions were assigned to functional networks as defined by Yeo et al. (Figure 4A,B). Permutation analysis showed that the DMN was significantly overrepresented among reported resilience findings (*p* < 0.001, permutation testing) (Figure 4C,D). Adapting the Yeo et al. parcellation to include a language network yielded significant results for both the DMN (*p* < 0.001) and language network (*p* = 0.006). These findings all survived FDR correction.

#### 3.2.3. Graph Theoretical Results

**Figure 5:**
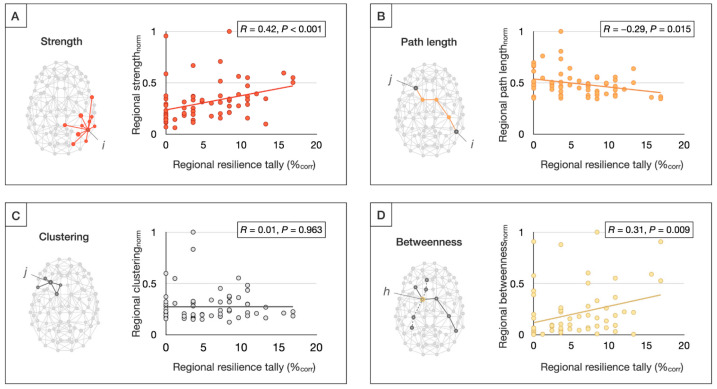
Graph theoretical meta-regression of resilience markers. Regional tallies of the resilience findings were examined for correlations with the metrics of brain network organization, including strength, reflecting the total sum of connectivity of a given node *i* (**A**); path length, computed as the average number of steps from any node *i* to any node *j* (**B**); clustering, signifying the average likelihood that two neighboring nodes of any node *j* were mutually connected (**C**); and betweenness centrality, reflecting the fraction of shortest paths in the network that contained a given node *h* (**D**). The network metrics were computed from weighted structural connectome reconstructions from a cohort of healthy controls from an independent study [35] and normalized between 0 and 1 for visualization purposes.

Regional tallies of resilience findings were positively correlated with regional connectivity strength (*r* = 0.42, *p* < 0.001) and betweenness centrality (*r* = 0.31, *p* = 0.009), and negatively correlated with the path length (*r* = −0.29, *p* = 0.015), suggesting that more highly connected, central, and efficient brain regions more commonly show resilience-related effects (Figure 5). Moreover, resilience-related findings were found to be significantly overreported among rich club hubs relative to non-rich club regions (*p* = 0.018). These findings also survived an FDR correction.

## 4. Discussion

This systematic review and label-based meta-analysis aimed to identify spatially consistent brain markers of resilience to psychosis across structural and functional MRI studies in (clinical and familial) high-risk cohorts and to assess potential underlying mechanisms. To the best of our knowledge, this is the first meta-analytical assessment of the neuroimaging literature on resilience to psychosis.

Our systematic review yielded a total of 43 neuroimaging studies that comprised almost five thousand participants and reported structural and functional brain changes associated with resilience to psychosis in at-risk individuals. Among the 35 studies that reported regionally specific findings, resilience-associated brain changes were found to be significantly overreported among the DMN, language network, and central brain hubs. Although regional findings did not survive multiple comparison corrections, overrepresented areas, including the precuneus and (medial) superior frontal gyrus, fusiform gyrus, and left IFG, converged largely on the same systems. The reported resilience-associated effects in these regions included increases in the volume, cortical thickness, or structural connectivity, and changes in the functional activation and connectivity. It remains to be determined how such brain changes would promote resilience to psychosis.

Two potential mechanisms promoting healthy brain and cognitive functioning include a higher brain reserve and compensatory neuroplasticity. The brain reserve has been defined as a higher quantity of neural resources acting as a buffer to subsequent pathological changes and thereby preserving normal functioning [37]. Often operationalized as a higher brain volume, the brain reserve has been associated with slower clinical deterioration in dementia [38,39], preserved cognition in superagers [40], and gains in cognitive performance after cognitive enhancement therapy for schizophrenia [41]. In the context of high-risk for psychosis, higher premorbid brain volume—globally or in specific regions—or “super-normal” levels of cortical thickness or structural connectivity may buffer an overshoot in synaptic pruning in adolescence, which is thought to contribute to the pathophysiology of psychotic illness [42] and thereby mitigate the disease process. In addition, compensatory neuroplasticity may perhaps underlie some of the resilience-related fMRI results reported in the literature. Changes in the functional activation or functional connectivity may, for example, result from brain regions that actively rewire through synaptic plasticity or inherent neuron excitability as a reciprocal response to changes in other areas [43,44]. Alone or in concert, these processes may play a role in shaping an individual’s capacity for resilience by buffering or offsetting risk-associated brain changes and thereby averting progression to full psychosis in at-risk youth.

The results of our label-based meta-analysis suggest that compensatory or adaptive changes of the DMN may be particularly beneficial to resilience. Our regional analysis yielded significant results for the precuneus and superior frontal gyrus, two important nodes of the DMN, with 10 out of 35 studies with region-specific results (28.6%) implicating either or both regions. Moreover, in our network-level analysis, the DMN was found to be significantly overrepresented among resilience-related results reported in the literature. The DMN’s involvement in psychosis is emphasized by studies showing abnormalities in task-activation and functional connectivity of the DMN in patients with schizophrenia, bipolar disorder, and CHR individuals [45,46,47,48,49,50]. In addition, DMN connectivity has been related to the clinical outcome in the at-risk stage [51,52]. Indeed, a PET study showed that adaptive plasticity of the MPFC, a key part of the DMN, may protect against psychosis development in the context of childhood trauma [53]. Overall, these findings suggest a central role for the DMN in both the risk of and resilience against psychosis. Interestingly, there is evidence that mindfulness-based interventions reduce the connectivity within the DMN [54]. These interventions may thus build resilience by ameliorating the aberrant DMN connectivity associated with psychosis [45]. Indeed, there is recent preliminary evidence that mindfulness-based resiliency training is effective in reducing symptoms among at-risk individuals [55]. Moreover, mindfulness-based real-time fMRI neurofeedback aimed at downregulating DMN showed promise in terms of reducing auditory hallucinations in schizophrenia patients [56]. Finally, preliminary preclinical evidence suggests that targeted early-stage neuromodulation of the medial prefrontal cortex may prevent brain and behavioral abnormalities associated with psychosis development [57], again suggesting that mPFC may be a valuable target for early intervention.

In addition to the DMN, the label-based meta-analysis suggested that the left IFG and larger language network may play a role in promoting resilience to psychosis. The IFG consists of orbital, triangular, and opercular parts and encompasses Broca’s speech area in the dominant (typically left) hemisphere. In regional analysis, a significant effect was found for the left orbital part of the IFG specifically, but 11 out of 35 studies with region-specific results (31.4%) reported that any part of the IFG showed resilience-related effects. In addition, a DWI study (Table A2) reported increased fractional anisotropy (FA) of the arcuate fasciculus in unaffected siblings of schizophrenia patients, while their affected relatives showed an association between arcuate fasciculus FA and symptom severity [58]. Connecting Broca’s area in the IFG to Wernicke’s area in the temporal cortex, the arcuate fasciculus is involved in speech and language processing [59,60,61] and has been implicated in auditory hallucinations [62]. Taken together, these findings suggest that increases in the cortical thickness of the IFG and/or increases in structural connectivity of the arcuate fasciculus connecting IFG to other language areas may attenuate risk for psychotic symptom development. This hypothesis is in line with evidence that language learning and bilingualism can build cognitive reserve and thereby protect against neuropsychiatric disorders [63,64].

Finally, graph-theoretical analysis showed that brain regions with high connectivity and efficiency (i.e., low pathlength) and topological centrality were more likely to be reported in the literature as showing resilience-related effects. In line with this observation, resilience markers were found to be overreported among rich club hubs. These graph theoretical findings are consistent with our regional and network-level results, as rich club hubs, including the precuneus, superior frontal gyrus, and superior parietal gyrus [36], show significant overlap with the DMN [65]. Moreover, the network results extend our regional findings by providing an additional mechanistic hypothesis on why these regions in particular may be beneficial to resilience: given their central role in global brain communication [66,67,68] and the disproportionate impairment of hub-to-hub connectivity observed in schizophrenia [35,69,70], brain hubs may be particularly well positioned to buffer risk-associated brain changes and thereby promote resilience to psychosis.

A number of possible limitations should be considered when interpreting the current results. First, our findings are based on a sparse literature that includes studies with modest sample sizes. We attempted to control for this issue by reducing the relative influence of findings from studies with small sample sizes or quality concerns, but well-powered, methodologically robust studies are needed to confirm our results. In addition, because of the paucity of literature on the neurobiology of resilience, all MRI studies, regardless of the imaging modality and at-risk definition, were included. An advantage of this approach is likely an increased sensitivity to detect putative resilience markers, as different at-risk groups may share risk- and resilience-related characteristics, and such changes may show up in different imaging modalities. A disadvantage is that including such a diverse set of studies precluded a more standard meta-analytical approach that could have yielded more robust findings. Moreover, although they largely matched with the network and graph theoretical results, the regional findings did not survive multiple comparison corrections and should thus be interpreted with caution. Therefore, we suggest that our findings are primarily used to generate novel hypotheses to be confirmed in future studies. In addition, the literature search that formed the basis for the current analysis was performed in January 2020, after which this study was unfortunately interrupted by the pandemic. Because of ensuing clinical obligations, it was not feasible for the research team to update the search to include studies up to 2024. As a result, the current findings may omit important studies that came out after 2020. Another potential limitation is that selected studies included mainly medicated patients, which may obscure the natural biology of the illness. As medication effects in patients can mimic resilience-related effects in high-risk individuals—lithium treatment, for example, has been linked to increased brain volumes, particularly in mood regulatory areas [71,72]—this could hinder the identification of brain markers of resilience. Antipsychotics were shown to mainly influence basal ganglia [73,74]. Given that we focused on cortical effects and on contrasts between high-risk individuals and both patients and healthy controls, it is unlikely that the currently reported results were confounded by the effects of antipsychotic medication. Moreover, given that the label-based analysis relied on tallying results from prior studies in which participant groups were mostly well-matched and scanned on the same magnet, factors such as age, sex, and scanner differences are not expected to drive the current results, although they were not separately assessed in the current study. Finally, our review was based largely on cross-sectional studies. Longitudinal studies are needed to confirm our findings and distinguish between static protective (i.e., brain reserve) and dynamic (i.e., compensatory neuroplastic) processes that promote resilience to psychosis.

## 5. Conclusions

In conclusion, the current results suggest that protective or adaptive changes in a specific set of neural systems, including DMN-related brain regions, language areas, the fusiform gyrus, and rich club hubs, may have a central role in resilience to psychosis. These observations are of interest as individual differences in these systems may help understand why some at-risk youth develop psychosis while others remain healthy. Moreover, identifying neural systems associated with resilience to psychosis may promote therapeutic innovation in early psychosis and the high-risk state; for example, by identifying novel targets for intervention, such as non-invasive brain stimulation or fMRI-assisted neurofeedback [56,75,76]. If such targeted intervention can induce resilience-associated brain changes, this may slow or prevent progression to psychosis in HR youth “In the hope that, by doing so, they can perhaps be inoculated against disorder” [77].

## Figures and Tables

**Figure A1:**
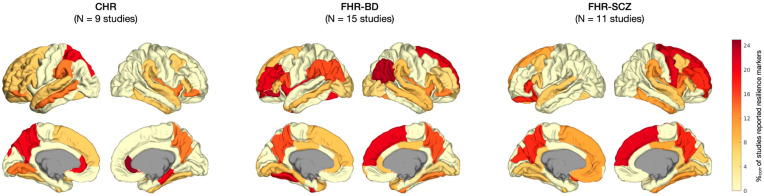
Patterns of resilience-associated finding per type of high-risk group. Cortical plots depicting localization of resilience markers per type of high-risk group: CHR versus FHR for bipolar disorder (FHR-BD) or FHR for schizophrenia (FHR-SCZ). Darker colors indicate more frequent reporting of resilience-associated effects within studies on this high-risk group. %_corr_ = corrected percentage of studies that reported resilience-associated effects.

## Data Availability

No new data were created. The tables and Supplementary Materials included in this publication contain all the data referenced in this study.

## Supplementary Materials

The following supporting information can be downloaded from https://www.mdpi.com/article/10.3390/brainsci15030314/s1, File S1: PRISMA 2020 Checklist; Figure S1. Language network definition; Supplementary results; List of Abbreviations (for Table A1, Table A2, Table A3 and Table A4); Table S1: Critical evaluation; Table S2. Mapped results—cortical; Table S3. Mapped results—subcortical; Table S4. DK atlas mapping details; Table S5. Studies excluded after full-text assessment, with rationale. References [78,79,80,81,82,83,84,85,86] are cited in the supplementary materials.

## Abbreviations

The following abbreviations are used in this manuscript:

CHR	Clinical high risk
DK	Desikan–Killiany (atlas)
DMN	Default mode network
FHR	Familial high risk
FG	Fusiform gyrus
HC	Healthy control
HR	High risk
IFG	Inferior frontal gyrus
MNI	Montreal Neurological Institute
MRI	Magnetic resonance imaging
PRISMA	Preferred Reporting Items for Systematic Reviews and Meta-Analysis
Rs-fMRI	Resting-state functional MRI

## Appendix A

**Table A1:** Anatomical MRI studies.

Paper	Participants Details	MRI Acquisition	MRI Analysis	Statistical Analysis	Main Resilience Findings
Fornito et al., 2008 [87]	35 resilient CHR (UHR-NP, 20 yr, 57% M) 35 non-resilient CHR (UHR-P, 19 yr, 60% M) 33 HC (21 yr, 64% M)	1.5T GE Signa: spoiled-GRE recoil sequence, TR 14.3 ms, TE 3.3 ms, FA 30°, FOV 24 cm, and voxel size 0.938 mm^2^ × 1.5 mm	Skull-stripping and alignment to N27 template with FSL; FreeSurfer for segmentation and cortical surface reconstruction.	ANOVA to test regional GM volume, SA, and CT; Bonferroni-adjusted α of *p* < 0.0167 (α/3) for 3 pairwise comparisons (i.e., HC, resilient CHR, and non-resilient CHR).	Resilient CHR showed increased the cortical thickness of the dorsal limbic ACC and a trend-level increase in the **rostral limbic ACC** compared with HC, and increased the thickness of the rostral limbic ACC and subcallosal paralimbic ACC (trend-level) compared with non-resilient CHR.
Habets et al., 2008 [88]	32 FHR (FDR, 36 yr, 44% M) 31 SCZ (31 yr, 48% M) 27 HC (36 yr, 44% M)	1.5T Philips Gyro-scan NT-I1: T2- and proton-density (PD)-weighted images (dual-echo FSE sequence, TR 4 s, TE_1_ 20 ms, TE_2_ 100 ms, FOV 22 cm, 60 slices, 3 mm thick, and interleaved, with 0.3 mm gap)	Brain mask generated from PD images, proportions of different tissues were determined for voxels within mask, and transformation of maps into standard space using AFNI.	ANCOVA modeling the effect of cognitive scores on GM volumes fitted for each voxel, permutation testing with β/SE(β) > 2 voxel threshold, and cluster thresholds limiting false positive tests to < 1 per map.	In addition to risk-associated cerebellar gray matter deficits shared with SCZ, FHR showed increased gray matter density of a cortical region, specifically the **SFG**. However, FHR showed a correlation between poorer executive performance and density of the SFG, cingulate gyrus, and cerebellum.
Kempton et al., 2009 [89]	50 FHR (FDR, 34 yr, 48% M, incl. 14 with MDD, 31 yr, 36% M) 30 BD (39 yr, 50% M) 52 HC (35 yr, 52% M)	1.5T GE Signa: T1 images (3D spoiled GRE sequence, TR 18 ms, TE 5.1 ms, FA 20°, and voxel size 0.94 mm^2^ × 1.5 mm)	SMP5 for VBM with unified segmentation.	Regional GM volumes were assessed for group effect using ANCOVA with ICV as the covariate, using a voxel-threshold of *p* < 0.001 (uncorrected) and cluster threshold ≥ 5.	BD patients, healthy FHR, and FHR with MDD all showed increased left insula volume (i.e., marker of BD risk); only healthy FHR showed an increased **left cerebellar** (**vermal**) volume compared with HC and BD, which is suggestive of an association with resilience.
Greenstein et al., 2011 [90]	80 FHR (sibs, 17 yr, 46% M) 94 COS (17 yr, 54% M) 110 HC (17 yr, 58% M)	1.5T GE Signa: T1 images (spoiled GRE sequence, TR 24 ms, TE 5 ms, FA 45°, FOV 24 cm, and contiguous 1.5 mm axial slices and 2.0 mm in the coronal plane)	MR images registered to standard space using linear transformation and BRAINS2 software package to parcellate the cerebellum.	Polynomial mixed model regression to assess the cerebellar development; *t*-tests at two time points (mean age, last scan after age 20) to assess group effects (alpha *p* < 0.01).	FHR and COS showed divergent trajectories of the total cerebellar volume; only adolescent FHR had a greater **superior vermis** volume. Across the follow-up (2 yr intervals up to 12 years), the developmental trajectory of the superior vermis in FHR converged with HC.
Frangou, 2012 ^$^ [91]	48 FHR (FDR, 37 yr, 50% M) 47 BD (46 yr, 45% M) 71 HC (40 yr, 51% M)	1.5T GE Neuro-optimized Signa: T1 scan (TR 1.8 s, TE 5.1 ms, TI 450 ms, FOV 4 × 18 cm, FA 20°, and voxel size 0.9 mm^2^ × 1.5 mm)	SPM5 for voxel-based morphometry using unified segmentation.	An ANCOVA model was used to assess the effect of the group on regional GM volumes, correcting for the ICV, with an uncorrected *p* < 0.001 voxel threshold and cluster threshold of 5.	In addition to putative risk markers (e.g., increased insula volume), FHR showed unique changes (relative to patients and HC), including a higher **cerebellar vermis** volume. Functional MRI findings from this study were also reported by Pompei et al., 2011 [92], as listed in Table A3.
van Erp et al., 2012 [93]	14 FHR (co-twins, 44 yr, 36% M) 18 BD (44 yr, 44% M, incl. 10 lithium-treated and 8 non-treated) 32 HC (twins, 47 yr, 53% M)	1.0T Siemens: T1 images (MPRAGE sequence, TR 11.4 ms, TE 4.4 ms, FOV 25 cm, and voxel size 0.98 mm^2^ × 1.2 mm)	Preprocessing using MNI tools (mritotal, N3). MultiTracer for hippocampal tracing; 3D radial distance mapping for hippocampal shape analysis and group comparison.	Mixed model regression to assess the hippocampal volume, SA, length, and thickness for group effects using permutation to assess the significance of statistical maps and correct for multiple comparisons.	Compared with controls, FHR co-twins showed a larger **hippocampal** thickness than controls along the border of the cornu ammonis and anterior subiculum, especially in the right hemisphere. These regions overlapped partly with areas that showed increased thickening in BD patients, possibly secondary to lithium-treatment mimicking resilience effects.
Eker et al., 2014 [94]	28 FHR (sibs, 35 yr, 40% M) 28 BD (36 yr, 58% M) 30 HC (35 yr, 33% M)	3.0T Siemens Magnetom Verio: T1 images (MPRAGE sequence, TR 1.6 s, TE 221 ms, TI 900 ms, FA 9°, FOV 25.6 cm, and voxel size = 1 mm^3^ mm). FLAIR and T2 scan to exclude brain lesions.	SPM8 for preprocessing and VBM analyses using DARTEL.	ANCOVA to assess group effects, correcting for age, sex, and ICV. Main analysis with FWE-corrected *p* < 0.05 and cluster > 5; exploratory analyses with uncorrected *p* < 0.001 and cluster > 50 in a priori selected areas.	In exploratory analyses, FHR had more gray matter in the **left DLPFC** than HC and BD. As this effect was unique to FHR and observed in the context of reduced gray matter in the left OFC in both FHR and BD, increased DLPFC gray matter density in FHR may reflect a compensatory response.
Chakravarty et al., 2015 [95]	71 FHR (sibs, 19 yr, 48% M) 86 COS (18 yr, 58% M) 81 HC (17 yr, 67% M)	1.5T GE Signa: T1 images (spoiled GRE sequence, TR 24 ms, TE 5 ms, FA 45°, FOV 24 cm, and axial contiguous 1.5 mm thick slices)	Striatum segmentation with MAGeT Brain algorithm; marching cubes method for surface reconstruction.	Shape measurements (vertex-wise) analyzed using mixed-model regression, adjusting for family ties using an FDR-corrected alpha.	FHR and COS both showed striatal shape changes, with outward displacement of the ventral striatum and inward displacement along the anterior head; in FHR, these **striatal shape abnormalities** normalized in early adulthood.
Goghari et al., 2015 [96]	26 FHR (FDR, 41 yr, 35% M) 25 SCZ (41 yr, 52% M) 23 HC (40 yr, 48% M)	3T GE Discovery MR750: T1 images (MPRAGE, TR 7.4 ms, TE 3.1 ms, FA 11°, FOV 25.6 cm, and 236 1 mm thick coronal slices)	FreeSurfer v5.1.0 for cortical reconstruction and parcellation (acc. to DK atlas).	ANCOVAs to assess group effects; univariate tests for lobes showing significant effects without further multiple comparison correction.	FHR showed greater cortical thickness of the **bilateral caudal MFG**, **IFG** (**opercular** and **triangular part**), **STG**, **isthmus-cingulate gyrus**, **precuneus**, **cuneus**, **LG**, **left FG, and lateral OFC** compared with HC and SCZ.
Sariçiçek et al., 2015 [97]	25 FHR (FDR, 32 yr, 46% M) 28 BD (36 yr, 26% M) 29 HC (34 yr, 28% M)	1.5T Philips Achieva: T1 images (FFE sequence, TR 25 ms, TE 6 ms, FA 8°, FOV 24 cm, axial slices, and 1 mm thickness)	VBM analyses in SPM8 using DARTEL.	ANCOVA for VBM group comparisons, with ICV and education as covariates; thresholds: cluster *p* < 0.05, voxel *p* < 0.01, and cluster size > 205.	Relative to HC, both FHR and BD had reduced volume of the cerebellum (incl. vermis) and increased volume of the bilateral IFG, but only FHR showed increased gray matter volume of the **left SMG and parahippocampal gyrus**.
Zalesky et al., 2015 [98]	86 FHR (sibs, 49% M) 109 COS (57% M) 102 HC (59% M) (age range 12 to 24 yr, mean age not reported)	1.5T GE Signa: T1 images (spoiled GRE sequence, TR 24 ms, TE 5 ms, FA 45°, FOV 24 cm, and contiguous 1.5 mm thick axial slices)	Neural net classifier to segment registered and corrected images, surface deformation algorithm, cortical thickness measured in native space, and network mapping on lobar and regional level (DK atlas).	Initial broad analysis to identify lobes with connectivity deficits, followed by localized approach focused on significant regions from the broad analysis. No further multiple comparison correction.	Risk-associated (i.e., shared with COS) deficits in CT correlations between the left occipital (pericalcarine gyrus and FG) and temporal (STG) lobes, normalized by mid-adolescence in FHR. Protracted adult-onset normalization in COS correlated with symptom improvement. In addition, FHR showed increased correlations between the right cingulate and right temporal and parietal lobes.
Chang et al., 2016 [99]	31 FHR (offspr, 18 yr, 68% M) 60 SCZ (18 yr, 48% M) 71 HC (21 yr, 38% M)	3.0T GE Signa HDX: T1 images (FSPGR sequence, TR 7.2 ms, TE 3.2 ms, FA 13°, FOV 24 cm, 176 slices, and voxels 1 mm^3^)	DARTEL in SPM8 for preprocessing, including segmentation, registration, and normalization to MNI template.	GM comparisons in full-factorial design, with age and sex as covariates and using voxel threshold of *p* < 0.01 in AlphaSim-corrected (cluster > 444 voxels), post hoc 2-sample *t*-tests.	FHR showed increased gray matter volumes of the **right cerebellum** (anterior and posterior lobe), **FG**, **ITG**, **SMG**, **and precentral gyrus** compared with both HC and SCZ (according to Table and Figure 2 as reported in Chang et al. (2016), text states differently).
de Wit et al., 2016 ^#^ [100]	16 resilient CHR (UHR-remitted, 15 yr, 76% M) 19 non-resilient CHR (UHR-non-remitted, 16 yr, 56% M) 48 HC (16 yr, 60% M) (ages at the baseline, with 6 year follow-up)	1.5T Philips: T1 images (FFE sequence, TR 30 ms, TE 4.6 ms, FA 30°, FOV 25.6 cm, and contiguous coronal slices of 1.5 mm)	FreeSurfer v5.1.0 for preprocessing and to compute the gray matter volume, CT, SA, and gyrification. Longitudinal FreeSurfer pipeline for between-session comparisons.	Effects of age, group, and their interaction were assessed using a linear mixed model; multiple comparison correction was not specified.	Resilient CHR showed an increased CT of the **bilateral caudal MFG and FG**; **left SFG**; **rostral MFG**; **orbital IFG**; **lateral OFC**; **MTG**; **banks of the STS**, **SMG**, **SPG**, **and precuneus**; **right ITG and parahippocampal gyrus;** and large volumes of the **left precuneus lateral OFC and pallidum**. There were also smaller decreases over time in the CT and volume of several areas.
Katagiri et al., 2018 [101]	37 resilient CHR (ARMS-N, 24 yr, 30% M, incl. 14 med naive) 5 non-resilient CHR (ARMS-P, 18 yr, 20% M) 16 HC (23 yr, 50% M)	1.5T Toshiba Excelart Vantage: T1 images (TR 24.4 ms, TE 5.5 ms, FA 35°, FOV 25 cm, and 35 sagittal slices of 2 mm)	FreeSurfer v5.2.0 with default processing settings for computing longitudinal corpus callosum volumes.	ANOVA testing CC sub-volume group effects; linear regression between longitudinal volume changes and symptoms (all *p* < 0.05, uncorrected).	While resilient CHR showed a reduction in the mid-anterior, central, and mid-posterior CCs at the baseline, subsequent volume increases in the **central CC** were associated with improvements in negative symptoms over the follow-up.
Katagiri et al., 2019 [80]	37 resilient CHR (ARMS-N, 24 yr, 30% M, incl. 14 med naive) 5 non-resilient CHR (ARMS-P, 18 yr, 20% M) 16 HC (23 yr, 50% M)	1.5T Toshiba Excelart Vantage: T1 images (TR 24.4 ms, TE 5.5 ms, FA 35°, FOV 25 cm, and 35 sagittal slices of 2 mm)	FreeSurfer v5.2.0 for preprocessing with standard settings for computing longitudinal striatal volumes.	ANOVAs testing striatal volume for group effects, and multiple regression to correlate the volume changes with symptoms (all *p* < 0.05, uncorrected).	There were no group differences in the baseline striatal volumes in resilient versus non-resilient CHR. Improvements in the positive symptoms were correlated with an increased **right nucleus accumbens** volume in resilient CHR.
Yalin et al., 2019 [102]	24 FHR (FDR, 32 yr, 46% M) 27 BD (36 yr, 37% M) 29 HC (33 yr, 38% M)	1.5T Philips Tesla Achieva: T1 images (FFE sequence, TR 8.7 ms, TE 4 ms, FA 8°, FOV 23 × 22 cm, and 1 mm thick slices)	FreeSurfer v5.3.0 for preprocessing and computing the regional cortical thickness and surface area (DK atlas).	Regional CT and SA group effects testedwith a GEE model to account for family ties, covarying for age, sex, and ICV (for SA), with a Bonferroni correction.	Exploratory analyses showed a significant increase in the right **STG** SA in FHR siblings relative to HC, whereas BD showed a trend-level increase in the SA of the STG (and a significant increase in the SA of the left IFG, triangular part).

Anatomical MRI studies reporting resilience-associated effects. Familial high-risk (FHR) individuals are first-degree relatives (FDRs) of schizophrenia (SCZ) or bipolar disorder (BD) patients; if the FHR group comprised offspring (offspr) or siblings (sibs) only, this is noted as such. For the clinical high-risk (CHR) groups, the specific risk syndrome (i.e., CHR, ARMS, UHR) is noted. ^$^ Frangou, 2012 results shown in this table include only the sMRI findings; task-fMRI results were also reported by Pompei et al., 2011 and can be found in Table A3. ^#^ de Wit et al., 2016 used two separate definitions of resilience; the results reported here are from the definition based on the criteria of remission defined by McGlashan et al. (2001) [103] to facilitate comparison with other literature. In the column “main resilience findings”, regions that showed resilience-related effects are noted in bold print. UHR-P/NP—UHR patients who developed/did not develop psychosis; ARMS-P/N—ARMS patients who developed/did not develop psychosis. ICV—intracranial volume; yr—average age in years. Other abbreviations in “List of Abbreviations” in the Supplementary Materials.

**Table A2:** Diffusion-MRI studies.

Paper	Participants Details	MRI Acquisition	MRI Analysis	Statistical Analysis	Main Resilience Findings
Hoptman et al., 2008 [104]	22 FHR (FDR, 20 yr, 32% M) 23 SCZ (37 yr, 70% M) 37 HC (23 yr, 46% M)	1.5T Siemens Vision: T1 scan (MRPAGE, TR 11.6 ms, TE 4.9 ms, FA 8°, FOV 30.7 cm, 172 slices, and voxels 1.2 mm^3^); DWI scan (TR 6 s, TE 100 ms, FOV 32 cm, b-value = 1000 s/mm^2^, 8 non-collinear gradients, NEX 7, voxels 2.5 mm^2^, 19 slices of 5 mm, and no gap)	FA calculated with in-house-developed software. T1-scans skull-stripped with FreeSurfer, registered to distortion corrected b = 0 images; b = 0 and FA maps transformed to Talairach space.	Voxelwise ANOVA with age and sex as covariates, extracting clusters of >100 contiguous voxels with *p* < 0.05 (incl. at least 1 voxel with *p* < 0.001). A lower threshold of 50 contiguous voxels was used in the follow-up analysis.	FHR showed an increased FA in the **left subgenual ACC**, **right MFG and SFG**, and **pontine tegmental white matter**, which may represent areas that offer protection against disease onset in those at familial high risk for psychosis.
Kim et al., 2012 [105]	22 FHR (FDR ^$^, 23 yr, 36% M) 15 SCZ (23 yr, 53% M) 26 HC (22 yr, 50% M)	1.5T Siemens Avanto: DWI scan (TR 5.9 s, TE 96 ms, FOV 23 cm, axial slices of 2 mm, no gap, voxels 1.8 mm^2^ × 4 mm, b-value 1000 s/mm^2^, and 12 non-collinear directions)	FSL preprocessing. Callosal boundaries traced with Moore–Neighbor algorithm, CC segmented into 200 equidistance surface points, and FA extracted from each.	FA of each surface point assessed for group effects with ANOVA, using age and sex as covariates and using random permutation with *p* < 0.01 to control the type 1 error rate.	While SCZ showed the decreased FA of CC splenium and genu (trend), FHR had an increased FA of the **genu**, which may reflect compensatory WM changes counteracting the influence of the genetic vulnerability to psychosis.
Boos et al., 2013 [58]	123 FHR (sibs, 27 yr, 46% M) 126 SCZ (27 yr, 80% M) 109 HC (27 yr, 50% M)	1.5T Philips Achieva: T1 scan (SPGR sequence, TR 30 ms, TE 4.6 ms, FA 30°, FOV 25.6 cm, 160–180 contiguous slices, and voxels 1 mm^2^ × 1.2 mm); DWI scan (32 diffusion-weighted volumes, b-factor 1000 s/mm^2^, 8 b = 0 volumes, TR 9.8 s, TE 88 ms, FA 90°, FOV 24 cm, 60 slices of 2.5 mm, and no gap)	DTI scans realigned, distortion-corrected, and transformed with ANIMAL software. Fiber reconstruction with FACT algorithm and the mean FA extracted per tract.	Mixed models to test 8 WM bundles for group effects in the FA while covarying for age, sex, handedness, interactions, and family ties; exploratory study without multiple comparison correction (results not significant after a correction).	FHR showed a higher FA of the **bilateral arcuate fascicles** relative to HC and SCZ, while the arcuate FA was negatively associated with symptom severity in SCZ. Together, these effects are suggestive of compensatory changes in the arcuate in FHR, which may guard against symptoms.
Goghari et al., 2014 [106]	24 FHR (FDR, 40 yr, 42% M) 25 SCZ (41 yr, 52% M) 27 HC (41 yr, 48% M)	3T GE: DWI scan (HARDI, 60 gradient directions with b-value 1300 s/mm^2^, and no other details provided)	ExploreDTI for preprocessing and deterministic tractography; seed-based tracking of fornix, in-house software for along-tract analysis.	Multiple ANCOVAs to assess the effects of group on the FA, MD, RD, and AD separately for fornix body and fimbria without multiple comparison correction; along-tract analyses were FDR corrected.	Along-tract analyses showed local increases in the FA in the **right fimbria of the fornix** in FHR compared with HC and SCZ, which may represent a compensatory mechanism to guard against psychosis.
Katagiri et al., 2015 [107]	34 resilient CHR (ARMS-N, 24 yr, 26% M, incl. 11 untreated) 7 non-resilient CHR (ARMS-P, 21 yr, 14% M) 16 HC (23 yr, 50% M)	1.5T Toshiba Excelart Vantage: DWI scan (single-shot EPI, TR 7.7 s, TE 100 ms, FOV 26 cm, voxels 1.02 mm^2^ × 5 mm, 30 axial slices along 6 gradient directions, b-value 1000 s/mm^2^, and unweighted b = 0 images)	Preprocessing in the FSL including distortion correction, masking, tensor fitting, and registration to standard space. TBSS for the between-group FA analysis.	Group comparisons with *t*-tests using randomize function in the FSL and threshold-free cluster enhancement method, with *p* < 0.05 and FWE correction for multiple comparisons.	At the baseline, CHR (relative to HC) showed a reduced FA of the left anterior CC. Resilient CHR showed improved subthreshold positive symptoms at one-year follow-up in association with an increase in the FA in the **left anterior CC (genu).**

Diffusion-MRI studies reporting resilience-related effects. See Table A1 legend for FHR and CHR criteria and abbreviations. In the column “main resilience findings”, regions that showed putative resilience effects are in bold print. ^$^ In Kim et al. (2012) [105], FDR had at least two third-degree relatives, including one first-degree relative with SCZ; yr—average age in years. Other abbreviations in “List of Abbreviations” in the Supplementary Materials.

**Table A3:** Task-based fMRI studies.

Paper	Participants Details	Task	MRI Acquisition	MRI Analysis	Statistical Analysis	Main Resilience Findings
**Working Memory (WM)**						
Fusar-Poli et al., 2010 [108]	15 CHR (ARMS, 24 yr, 53% M, incl. 13 resilient CHR) 15 HC (25 yr, 60% M)	Paired association learning task	1.5T Signa (GE): T2 * scan (no MR sequence reported, TR 2 s, TE 40 ms, FA 90°, 14 axial planes, 38 slices of 3 mm, and 0.3 mm gap) and high-res IR-prepped dataset (TR 1.6 s, TE 80 ms, and TI 180 ms)	Processing in SPM5; functional volumes realigned to the first volume and corrected for motion artifacts (no further details).	Full factorial model 2nd-level analysis to assess the cognitive load, group, and interaction effects. Pairwise *t*-tests to assess the longitudinal changes with voxel-wise threshold *p* < 0.05 with FWE correction.	At baseline, hypoactivation of the left precuneus, SPF, and MTG was observed in CHR, as well as a failure to activate parietal areas with increasing task difficulty. Improved clinical status at follow-up correlated with a longitudinal compensatory activation increase in the **left LG and SPL**.
Fusar-Poli et al., 2011 [109]	15 CHR (ARMS, 24 yr, 53% M, incl. 13 resilient CHR) 15 HC (25 yr, 60% M)	N-back task (0-, 1-, or 2-back)	1.5T Signa (GE): T2 * scan (GRE sequence, TR 2 s, TE 40 ms, FA 90°, 14 axial slices of 3 mm, and 0.3 mm gap); high-resolution inversion recovery dataset (TR 1.6 s, TE 80 ms, and TI 180 ms); T1 scan (SPGR sequence, TR 0.3 s, FA 20°, and 128 axial slices of 1.5 mm)	Functional images with SPM5; T1 scan with VBM5. Biological para-metric mapping for VBM-fMRI integration. Volumes corrected for motion artifacts, with no further details.	2nd-level analysis to test group effects in task activation using independent sample *t*-test, with whole-brain voxel-wise *p* < 0.05 and a FWE correction. Pairwise *t*-tests to explore longitudinal change and assess the effect of the functional outcome.	At baseline, CHR showed reduced task-related activation of the MFG, SMG, and IPL and lower GM volumes of the middle and medial frontal gyri, insula, and ACC. Between the baseline and follow-up, CHR showed longitudinal increase in activation of the **right parahippocampal gyrus and ACC**, which was correlated with functional improvement.
Choi et al., 2012 [110]	17 FHR (FDR ^#^, 21 yr, 53% M) 21 CHR (UHR, 22 yr, 57% M) 15 SCZ (23 yr, 53% M) 16 HC (21 yr, 56% M)	Spatial delayed-response task	1.5T Siemens Avanto: functional images (multi-slice EPI, TR 2.34 s, TE 41 ms, FA 90°, FOV 21 cm, and 25 axial interleaved slices); T1 scan for co-registration and anatomical localization (176 contiguous axial slices, with no other details)	Preprocessing in SPM2; volumes realigned to correct for interscan movement and stereotactically normalized.	2-sample *t*-test for between-group analysis with uncorrected voxel threshold *p* < 0.001, with cluster size > 15. Correlation analysis with behavioral performance and clinical variables.	FHR showed higher activity of **DLPFC** (BA9), **VLPFC** (BA 44), and **left thalamus** during WM encoding and maintenance. CHR showed a negative correlation between the thalamus activity and symptoms. Increased WM-related activation of PFC and thalamus may constitute a compensatory mechanism in FHR.
Smieskova et al., 2012 [111]	16 resilient CHR (ARMS-LT, 25 yr, 69% M) 17 non-resilient CHR (ARMS-ST, 25 yr, 77% M) 21 SCZ (29 yr, 76% M) 20 HC (27 yr, 50% M)	N-back task (0-, 1-, and 2-back)	3T Siemens Magnetom Verio: functional images (EPI sequence, TR 2.5 s, TE 28 ms, FOV 22.8 cm, voxels 3 mm^3^, 38 slices, 0.5 mm gap, and 126 volumes) and T1 scan (MPRAGE, TR 2 s, TE 3.4 ms, voxels 1 mm^3^, and TI 1 s)	Processing in SPM8 for functional and VBM8 for structural images. Functional volumes realigned to the first volume and corrected for motion artifacts.	ANCOVA to assess effect of group on task activation, covarying for age, sex, and voxel-wise GMV to assess significance as the cluster level using random-field theory (threshold *p* < 0.05 with an FWE correction).	Resilient CHR (i.e., CHR-LT) had a higher activation of **bilateral precuneus** and **right IFG**/**insula** than SCZ and CHR-ST, with an intact N-back performance and reaction times. Insular and IFG activation were associated with GM volumes in these regions in CHR-LT and may thus reflect resilience-related processes.
Stäblein et al., 2018 [112]	22 FHR (FDR, 43 yr, 36% M) 25 SCZ (37 yr, 68% M) 25 HC (35 yr, 48% M)	Masked change detection task	3T Siemens Magnetom: T2 * scan (GRE-EPI, TR 2 s, TE 30 ms, FA 90°, FOV 19.2 cm, voxels 3 mm^3^, 30 slices with 0.6 mm gap, and 456 volumes in 2 runs during 1 session); and T1 scan for co-registration (MPRAGE, 160 sagittal slices, TR 2.25 s, TE 2.6 ms, FA 9°, FOV 25.6 cm, and voxels 1 mm^3^)	BrainVoyager QX v2.8.4; 3D head motion correction; datasets with motion exceeding 3 mm in each direction were discarded.	2nd-level random effects repeated measures ANOVA; statistical maps FDR-corrected with cluster size > 160 and voxel threshold *p* < 0.01; Monte-Carlo simulation to assess cluster-level type 1 errors with *p* < 0.05 false positive rate.	FHR showed an increased **right insula and precentral gyrus** activity without behavioral deficits and a shift from decreased frontal activity at short intervals to increased activity at longer intervals, suggesting that WM consolidation may have been slowed in FHR, which allowed for the deployment of compensatory neuronal resources during encoding to support the WM performance.
**Cognitive Control**						
Pompei et al., 2011 [92]	25 healthy FHR (FDR, 35 yr, 52% M) 14 depressed FHR (FDR, 31 yr, 36% M) 39 BD (39 yr, 49% M) 48 HC (36 yr, 52% M)	Stroop Color Word Test (SCWT)	1.5T GE Neuro-optimized Signa: T2 * (EPI sequence, TR 3.5 s, TE 40 ms, FA 90°, voxels 3.75 mm^2^ × 7 mm, 18 non-contiguous axial slices, and 0.7 mm gap); T1 scan (TR 1.8 s, TE 5.1 ms, TI 450 ms, FOV 4 × 18 cm, FA 20°, and voxels 0.9 mm^2^ × 1.5 mm)	SPM 5 for preprocessing and PPI analysis during SCWT; motion correction methods not reported.	One-sample *t*-test random effects analysis (*p* < 0.0001 voxel and *p* < 0.05 cluster thresholds) to compute the contrast images from the within-group PPI analysis. Interaction between the PPI and group analyzed to test the group effects.	Alongside putative risk markers, resilient FHR showed increased decoupling between the **right VLPFC** and **bilateral insula** and additional coupling between the **right VLPFC** and **bilateral DLPFC**, which was hypothesized to reflect an adaptive functional change associated with resilience to BD.
**Emotion Recognition/Processing**						
Spilka et al., 2015 [113]	27 FHR (FDR, 41 yr, 37% M) 28 SCZ (41 yr, 54% M) 27 HC (41 yr, 48% M)	Passive facial emotion perception task	3T GE Discovery MR750: fMRI (EPI sequence, TR 2.5 s, TE 30 ms, FA 77°, FOV 22 cm, and 40 slices of 3.4 mm); T1 scan (TR 7.4 ms, TE 3.1 ms, TI 650 ms, FOV 25.6 cm, and 236 1 mm slices)	FSL v5.0.6 for preprocessing and analysis; motion parameters included as regressors of non-interest.	Subject-specific effects into mixed-effects model using unpaired *t*-tests for between-group comparisons, with a voxel threshold of *z* > 2.3 and cluster *p* < 0.05 with an RFT correction.	In addition to the hypoactivation of face-processing areas, FHR showed hyperactivation of frontal emotion-processing areas (**left triangular IFG and OFC**), possibly reflecting compensatory cortical recruitment to maintain intact facial emotion perception.
Sepede et al., 2015 [114]	22 FHR (FDR, 32 yr, 32% M) 23 BD (35 yr, 39% M) 24 HC (33 yr, 33% M)	IAPS-based emotional task (identifying vegetable items inside neutral or negative pictures)	1.5T Philips Achieva: T2 * (EPI sequence, TR 3 s, TE 50 ms, FA 90°, voxel size 4 mm^3^, 30 transaxial slices, and no gap); T1 scan (3D sequence, TR 25 ms, TE 4.7 ms, FA 30°, and voxels 1 mm^3^)	BrainVoyager QX 2.2 for processing; motion correction as part of pre-processing, with no details provided.	Group effects tested with random effect GLM, controlling for performance and mood symptoms and using voxel *p* < 0.001 and cluster > 4 to account for multiple comparisons.	BD patients showed reduced accuracy in target detection, while FHR performed similar to HC. Compared with both HC and BD, FHR showed hyperactivation of **right LG** and reduced activation of **right SFG and pre-SMA**; may reflect resilience markers.
Tseng et al., 2015 [115]	13 FHR (FDR, 14 yr, 62% M) 27 BD (14 yr, 56% M) 37 HC (15 yr, 43% M)	Face encoding task	3T GE: T2 * (single-shot EPI-GRE, TR 2 s, TE 40 ms, FOV 24 cm, voxels 3.75 mm^2^, and 23 contiguous slices of 5 mm); T1 (MPRAGE, TR 11.4 ms, TE 4.4 ms, TI 300 ms, FOV 25.6 cm, and 180 1 mm sagittal slices)	Preprocessing using SPM8 including motion (no details provided), slice timing correction, and normalization to MNI space.	Whole-brain ANOVA with group as the between-subject variable, using *p* < 0.001 (uncorrected) and cluster size > 10, without further mention of multiple comparisons.	Both BD and FHR showed hypo-activation of the left MFG during correctly vs. incorrectly recognized faces, but BD showed additional hypoactivation while FHR showed hyperactivation of the **right parahippocampal gyrus**, suggesting a possible compensatory process.
Dima et al., 2016 [116]	25 FHR (FDR, 40 yr, 54% M) 41 BD (44 yr, 52% M) 46 HC (40 yr, 49% M)	Facial affect recognition paradigm	1.5T GE Sigma: T2 * images (no MR sequence reported, TR 2 s, TE 40 ms, FA 70°, voxels 3.75 mm^2^ × 7.7 mm, and 450 volumes) and T1 scan (IR-prepped SPGR sequence, TR 18 ms, TE 5.1 ms, FA 20°, and voxels 0.94 mm^2^ × 1.5 mm)	SPM8 for preprocessing, conventional fMRI analysis, and DCM analysis; no motion correction reported.	Group effects tested using ANOVA with symptoms score as the covariate using FWE-corrected *p* < 0.05 and cluster size > 20; ANOVA or Kruskal–Wallis used to test the DCM output.	During face affect recognition, both BD and FHR showed higher fronto-limbic connectivity, but only FHR showed additional hyperconnectivity between the **FG** and **IOG**, suggesting additional recruitment in the affect-processing network as an adaptive neural response to emotional faces.
Welge et al., 2016 [117]	32 healthy FHR (offspr, 15 yr, 28% M) 32 depressed FHR (offspr, 14 yr, 19% M) 32 BD (16 yr, 41% M) 32 HC (15 yr, 34% M)	Continuous performance task with emotional and neutral distractors (CPT-END)	4.0T Varian Unity INOVA: T2 * images (GRE-EPI, TR 3 s, TE 29 ms, FOV 20.8 cm, FA 75°, and 5 mm thick slices) and T1 scan for anatomical localization (30 contiguous axial slices of 5 mm, with no further details)	Preprocessing and analysis in AFNI; small movement corrected via realignment; excessive motion or warped volumes removed.	A Bayesian hierarchical model was used to limit type 1 errors for pairwise comparisons between 4 clinical groups in 16 predefined ROIs.	All FHR showed greater task-related activation in left BA 44 (**IFG, opercular part**) relative to HC and healthy FHR showed higher activation in right BA 10 (**FP**) relative to BD and depressed FHR, possibly reflecting a compensatory response relevant to resilience to BD.
Spilka and Goghari, 2017 [118]	27 FHR (FDR, 41 yr, 37% M) 28 SCZ (41 yr, 54% M) 27 HC (41 yr, 48% M)	Facial emotion discrimination under a target emotion condition; age discrimination task	3T GE Discovery MR750: T2 * (GRE sequence, TR 2.5 s, TE 30 ms, FOV 22 cm, voxels 3.4 mm^3^, 40 interleaved slices, and 206 vols); T1 (MPRAGE, TR 7.4 ms, TE 3.1 ms, FOV 25.6 cm, and 236 1 mm slices)	Preprocessing and analysis in FSL v5.0.6; time-series plots of estimated head motion were inspected, with >3.5 mm excluded.	Between-group comparisons using unpaired non-parametric *t*-tests, with a voxel *p* < 0.001 threshold and FWE-corrected *p* < 0.05 cluster threshold.	FHR showed higher deactivation of **bilateral precuneus** and **right PCC** during age discrimination and of **left cuneus** during emotion discrimination, possibly reflecting inhibition of internally generated thought to maximize external attention toward task stimuli.
Wiggins et al., 2017 [119]	22 FHR (FDR, 16 yr, 59% M) 36 BD (18 yr, 58% M) 41 HC (17 yr, 51% M)	Face Emotion Labeling Task (identifying emotions on faces with different intensities of emotions)	3T GE MR750: fMRI (single-shot EPI-GRE, TR 2.3 s, TE 25 ms, FA 50°, FOV 24 cm, voxel size 2.5 mm^2^ × 2.6 mm, and 47 contiguous axial slices); T1 scan for spatial normalization (FA 15°, FOV 24 cm, and 124 axial slices of 1.2 mm)	AFNI for processing and mixed model analysis. Motion parameters included in the baseline model. TR pairs with the >1 mm frame-wise displacement censored.	Whole-brain linear mixed model with group as between-subjects factor and emotion/intensity as within-subject factors using voxel a *p* < 0.005 threshold and cluster ≥ 39, equivalent to an FDR-corrected *p* < 0.05.	In addition to changes shared with BD (risk markers), FHR showed hyperactivation of the **bilateral PCC/precuneus**, **IFG**, **SFG, temporo-parietal areas**, **TP/insula,** and **left FG** and hypoactivation of the **left angular gyrus**; this may reflect increased neural sensitivity to social cues by compensating for deficits in executive areas.
Nimarko et al., 2019 [120]	27 resilient FHR (BD offspr, 13 yr, 56% M) 23 non-resilient FHR (BD offspring, 14 yr, 30% M) 24 HC (15 yr, 42% M)	Implicit emotion perception task (viewing images of happy, fearful, or calm expressions)	3T GE Signa: fMRI (spiral in–out pulse sequence, TR 2 s, TE 30 ms, FA 80°, FOV 22 cm, 30 axial slices of 4 mm, and 1 mm gap); T1 scan for normalization (FSPGR sequence, TR 8.5 ms, TE 3.32 ms, TI 400 ms, FA 15°, FOV 25.6 cm, and 186 axial slices of 1 mm)	FSL featuring motion correction with MCFLIRT if mean displacements > 2 mm or more than 1/3 of the volumes had DVARS values > 75th percentile plus 1.5 times the interquartile range.	Group effects tested with whole-brain voxel-wise *t*-tests corrected for age and sex using voxel threshold *z* > 2.3 and cluster *p* < 0.05 and corrected for multiple comparisons.	Resilient FHR showed **right precuneus** and **left IFG** hypoactivation relative to non-resilient FHR, and higher connectivity between the **left IFG** and the **left MFG, MTG, and insula** for fear > calm contrast, and between the **left IPL** and **left precuneus/LG**, and the **right SMG and left FG** for happy > calm, which was associated with improved pro-social behavior and functioning.
**Theory of Mind**						
Brüne et al., 2011 [121]	10 CHR ^$^ (26 yr, 70% M, incl. 1 converter at 1yr follow-up) 22 SCZ (27 yr, 68% M) 26 HC (29 yr, 64% M)	Theory of mind task	1.5T Siemens Magnetom Symphony: functional images (single-shot EPI, TR 3 s, TE 60 ms, FOV 22 cm, voxels 3.5 × 3 mm^3^, FA 90°, 30 trans-axial slices, 0.3 mm gap, and 157 scans; T1-scan (MPRAGE, TR 1.8 s, TE 3.87 ms, FOV 25.6 cm, voxels 1 mm^3^, and 160 sagittal slices)	Preprocessing and analysis in SPM5 using MarsBaR toolbox to derive ROIs; no motion correction reported.	Second-level analysis to locate ToM regions with uncorrected *p* < 0.05 and cluster size > 10. Group effects in ToM-area activation assessed with two-sample *t*-test with *p* < 0.05 and cluster size > 10.	CHR activated the ToM network (PFC, PCC, and temporoparietal cortex) more strongly than SCZ and (in part) HC. Specifically, CHR showed increased activation of the **left IFG**, **bilateral STG and SMG**, **left MTG, and HG**. This may suggest the compensatory overactivation of brain regions critical for empathic responses during mental state attribution.
Willert et al., 2015 [122]	21 FHR (FDR, 31 yr, 33% M) 24 BD (45 yr, 50% M) 81 HC (36 yr, 49% M)	Theory of mind task	3T Siemens Trio: functional images (EPI sequence, TR 2 s, TE 30 ms, FA 80°, FOV 19.2 cm, 28 slices of 4 mm, and 240 volumes)	Preprocessing in SPM8 with gPPI (generalized form of context-dependent PPI), with 6 regressors modeling head motion included in 1st-level analyses.	ANCOVAs to test the activation and connectivity of 4 predefined ROIs corrected for age, sex, education, and task response, with an FWE correction for the number of ROIs.	BD patients showed reduced TPJ activation and reduced fronto-TPJ connectivity, while FHR showed increased activation of the **right MTG** and stronger connectivity between the **right MTG** and **MPFC**, suggesting compensatory MTG recruitment during mental state attribution in FHR.

* Reported resilience effects from task-fMRI studies, organized according to the task paradigm. See Table A1 legend for FHR and CHR criteria and abbreviations. The “main resilience findings” column lists regions with putative resilience effects in bold print. ^#^ FHR in Choi et al. (2012) had one first-degree or two second-degree relatives with SCZ. ^$^ The specific risk syndrome in Brüne et al. (2011) [121] was unclear. ARMS-LT/ST—ARMS–long-term/short-term; MDD—major depressive disorder; yr—average age in years. Other abbreviations in the Supplementary Materials.

**Table A4:** Resting-state fMRI studies.

Paper	Participants Details	MRI Acquisition	MRI Analysis	Statistical Analysis	Main Resilience Findings
Anticevic et al., 2014 [123]	21 FHR (offspr, 20 yr, 47% M) 48 SCZ (28 yr, 44% M, incl. 20 chronic and 28 early course) 96 HC (29 yr, 45% M)	3T GE Signa HDX: T2 * images (GRE-EPI, TR 2 s, TE 30 ms, FOV 24 cm, 35 axial slices of 3 mm, and 200 volumes) and T1 scan (FSPGR, TR 7.1 ms, TE 3.2 ms, FA 13°, FOV 24 cm, 176 slices of 1 mm, and no gap)	FreeSurfer to segment amygdala seed; group comparisons of connectivity seed maps with FSL; rigid-body motion correction, volumes with a single FD > 1 functional voxel were excluded.	2nd-level ANOVA to assess the effects of the group, with whole-brain type 1 error correction via threshold-free cluster enhancement using 10,000 permutations.	While the SCZ patients showed reduced amygdala connectivity with the OFC, FHR showed increased connectivity between the **amygdala** and a **brainstem** region around the noradrenergic arousal nuclei implicated in stress responses, which may reflect either a risk or resilience mechanism in young FHR.
Guo et al., 2014 [124]	28 FHR (sibs, 26 yr, 54% M) 28 SCZ (23 yr, 54% M) 60 HC (27 yr, 58% M)	1.5T GE Signa Twinspeed: functional images (GRE-EPI, TR 2 s, TE 40 ms, FA 90°, FOV 24 cm, 20 transverse slices of 5 mm, and resolution of 3.75 mm^2^)	Preprocessing using SPM8 and DPARSF; functional scans realigned to the middle volume; head motion parameters were regressed out of the data.	One-way ANOVA for group effects in mean connectivity and distance; connectivity strength of 4005 node pairs tested using Bonferroni-corrected independent *t*-tests to localize the strongest effects.	FHR and SCZ showed proportional decreases in long-range relative to short-range connectivity, but only FHR showed the strengthening of existing long-range links, which is suggestive of a compensatory process. Moreover, FHR showed the higher strength of the short- and long-range salience network, short-range subcortical, and long-range frontal network (trend-level) connections.
Doucet et al., 2017 [125]	64 FHR (sibs, 32 yr, 42% M) 78 BD (34 yr, 33% M) 41 HC (33 yr, 32% M)	3T Siemens Allegra: T2 * images (single shot GRE-EPI, TR 1.5 s, TE 27 ms, FOV 24 cm, FA 60°, and 3.43 × 5 mm^3^ voxel size); T1 scan (MPRAGE, TR 2.2 s, TE 4.13 ms, TI 766 ms, FA 13°, and voxel 0.8 mm^3^)	Preprocessing with SPM12 and REST toolbox; graph analyses with Brain Connectivity Toolbox; average motion regressed from graph metrics.	Permutation analysis to assess group effects in global connectivity and modularity metrics at an FDR-corrected *p* < 0.05 and regional degree and participation using *p* < 0.05 following permutation testing.	BD and FHR showed lower cohesiveness of sensorimotor network, with associated reduction in integration of DMN regions (MPFC, hippocampus) in BD, while FHR showed increased participation coefficients of the **ventral ACC**, **angular gyrus**, **and SMA** and the nodal degree of the **L IFG (orbital part),** suggesting possible resilience markers.
Duan et al., 2019 [126]	89 FHR (FDR, 25 yr, 58% M) 137 SCZ (24 yr, 39% M) 210 HC (26 yr, 38% M)	3T GE Signa HD: functional images (GRE-EPI, TR 2 s, TE 30 ms, FA 90°, FOV 24 cm, 35 slices of 3 mm, and no gap)	Preprocessing SPM12 and DPARFS; PAGANI toolkit for network reconstruction and analyses; removed subjects with excessive motion (>3 mm or 3°).	ANCOVA to test group effects, with age and sex as covariates and an FDR-corrected *p* < 0.05 or voxel-wise *p* < 0.001 and *p* < 0.05 cluster threshold, with an RFT correction.	SCZ showed increased medium- and long-range distance strengths of the orbital IFG, while FHR showed a reduced distance strength of this region, possibly representing an adaptive response to maintain segregation/integration balance of the functional brain network in FHR.
Ganella et al., 2018 [127]	16 FHR (FDR, 58 yr, 13% M) 42 SCZ (41 yr, 70% M, i.e., treatment resistant) 42 HC (39 yr, 59% M)	3T Siemens Avanto Magnetom TIM Trio: T2 * images (EPI sequence, TR 2.4 s, TE 40 ms, and voxel size 3.3 × 3.5 mm^3^) and T1 scan (MPRAGE, TR 1.98 s, TE 4.3 ms, FA 15°, FOV 25 cm, and 176 sagittal slices of 1 mm)	Preprocessing using FSL and SPM8; head motion controlled with Friston 24-parameter model; rs-volumes with FD > 0.5 mm excluded.	ANCOVA analysis with age and sex as covariates was used to test each pairwise connection for group effects, with the NBS analysis using a *p* < 0.01 primary threshold, and an FWE-corrected *p* < 0.05 sub-network threshold.	Functional connections that showed group differences were classified as resilience, risk, or illness related. A minority (~5%) of connections classified as resilience mainly involved reduced connectivity between temporal (i.e., **TP**) and subcortical regions (**posterior cingulum**).
Guo et al., 2020 [128]	28 FHR (FDR, 26 yr, 54% M) 28 SCZ (25 yr, 54% M) 60 HC (27 yr, 58% M)	1.5T GE Signa Twinspeed: functional images (GRE-EPI, TR 2 s, TE 40 ms, FA 90°, and FOV 24 cm) and T1 scan (20 contiguous 5 mm thick transverse slices with 1 mm gap)	Preprocessing with SPM8 and DPARSF; variance explained by head motion differences was removed in primary and secondary correlation analyses.	ANOVA was used to assess group effects on connectivity and graph metrics using an FDR-correction with post hoc pairwise *t*-tests, correcting for age, sex, and motion effects.	FHR demonstrated greater global functional connectivity diversity than HC and SCZ, and a higher level of global degree, clustering coefficient, and global efficiency compared with the other groups.

* Resting-state fMRI studies reporting resilience-associated effects. See Table A1 legend for FHR and CHR criteria. In the “main resilience findings” column, regions associated with putative resilience-effects are in bold print. yr—average age in years. Other abbreviations in Supplementary Materials.

## Appendix B

### Appendix B.1. Summary of Resilience-Associated Findings in Subcortical Structures

Multiple studies reported putative resilience-related effects of subcortical areas. These results were not further assessed statistically but are summarized below. Details on individual studies can be found in Table A1, Table A2, Table A3 and Table A4.

#### Appendix B.1.1. Cerebellum

Several papers implicated the cerebellum in relation to resilience to psychosis. Chang et al. (2016) found an increased cerebellar gray matter density, specifically of the anterior and posterior lobes in resilient FHR as compared with HC and schizophrenia patients [99]. In addition, three studies reported larger volumes of the cerebellar vermis in resilient FHR compared with patients and HC [91,93,94].

#### Appendix B.1.2. Corpus Callosum

In addition, resilience-associated changes of the corpus callosum (CC) were observed in two high-risk cohorts: Kim et al. (2012) found an increased FA of the genu in the first-degree relatives of schizophrenia patients, while patients showed a reduced FA of the splenium [107]. Katagiri et al. (2015) reported that resilient CHR individuals showed an increase in the FA of the anterior CC from the baseline to the one-year follow-up that was correlated with improved subthreshold symptoms [107]. In another study in the same cohort, volume increases in the central CC were found to correlate with improvements in negative symptoms [101].

#### Appendix B.1.3. Basal Ganglia, Thalamus, Hippocampus, and Amygdala

Finally, five studies reported resilience-associated changes in the basal ganglia, thalamus, amygdala, and hippocampus [95,100,103,110,125]. These results did not converge on any one specific subcortical area.
