# Supplementary material for: Brain Markers of Resilience to Psychosis in High-Risk Individuals: A Systematic Review and Label-Based Meta-Analysis of Multimodal MRI Studies
Source: Brain Sci. 2025 Mar 17;15(3):314. doi: 10.3390/brainsci15030314 (PMC11939873; doi:10.3390/brainsci15030314)
Supplement: Supplementary file 1 [file brainsci-15-00314-s001.zip › brainsci-3476902-Collinetal_BrainSciences_SupplementaryMaterial.pdf]

Supplementary Material

Brain Markers of Resilience to Psychosis in High-Risk Individuals: A Systematic Review and Label-Based Meta-Analysis of Multimodal MRI Studies

Guusje Collin [1,2,3,*], Joshua E. Goldenberg [4], Xiao Chang [5,6], Zhenghan Qi [3,7,8], Susan Whitfield-Gabrieli [9], Wiepke Cahn [10,11], Jijun Wang [12], William S. Stone [13], Matcheri S. Keshavan [13] and Martha E. Shenton [4,14]

Supplementary methods. Critical evaluation, regional mapping, network-level analysis

Figure S1. Plot of language network definition as used in systems-level follow-up analysis.

List of abbreviations. Abbreviations used in Tables 1 – 4 in main text and Table S3.

References. Reference list for citations in supplementary materials.

Table S1. Critical evaluation.

Table S2. Mapped results – Cortical Left and Right Hemisphere

Table S3. Mapped results – Subcortical (Bilateral).

Table S4. DK-atlas mapping details.

Table S5. Studies excluded after full-text assessment, with rationale

Supplementary methods

Critical evaluation: quality adjustment

Given that the literature on resilience markers is sparse, studies with quality concerns were not simply excluded. Rather, their findings were weighted according to the number of potential quality issues. Three specific quality concerns were assessed for each study: 1) sample size (i.e., minimum of 20 participants per group or 60 participants in total); 2) adequate motion correction; and 3) appropriate multiple comparison correction. To mitigate the influence of potential quality issues, less weight was assigned to findings from studies with quality concerns: i.e., studies with one or two quality concerns were weighted 0.67 and 0.33 respectively (Table S1 provides details on quality-assessment of each included study). Weighted results were used to compute regional and system-level tallies of resilience findings as reported in the main text (Table S2 shows quality-weighted results for cortical brain regions; Table S3 shows subcortical results).

Regional analysis: mapping to Desikan-Killiany atlas

This systematic review incorporates findings from studies using varying imaging modalities and processing methods. In order to assess whether reported resilience-markers (irrespective of MRI modality and processing methodology) converged on one or more specific areas of the brain, a region-wise meta-analysis was performed. To this end, findings were mapped to the Desikan-Killiany (DK) atlas. Results from studies using the DK atlas or a similar atlas were transferred directly. Results from studies using a different atlas or a voxel-based method, were mapped to DK-atlas using MNI coordinates or by close examination of applicable figures by two authors (GC and JEG). For mapping details of each included study, see Table S4.

Network-level analysis: language network

In the network-level analysis, regions of the DK-atlas were decomposed into seven networks according to Yeo et al. (2011). The standard Yeo et al. parcellation does not include a language network (Yeo et al., 2011). Given the known importance of language-related symptoms such as auditory verbal hallucinations and formal thought disorder to schizophrenia spectrum disorders and the high-risk state (DeVylder et al., 2014; Levy et al., 2010; Strik et al., 2008) and their associations with abnormalities in language areas (Ćurčić-Blake et al., 2017; Vita et al., 1995), we performed a follow-up analysis in which the Yeo et al. parcellation was adapted to include a

language network. The language network used in the current study was modeled after the language network from the CONN toolbox (Nieto-Castanon, 2020; Whitfield-Gabrieli and Nieto-Castanon, 2012), which is based on an Independent Component Analysis of fMRI data from the Human Connectome Project, as mapped to DK-atlas (Figure S1).

Figure S1. Language network definition

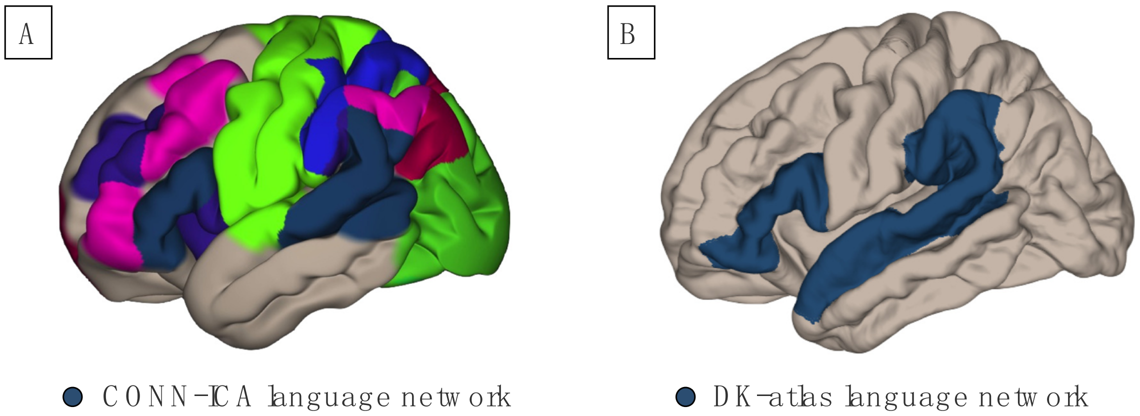

Figure legend: Language network definition (in dark blue) based on ICA analysis of HCP data (A), mapped to DK-atlas (B), reflecting the language network definition used in the current study.

List of Abbreviations

Tables 1 – 4 in the main text include several abbreviations pertaining to MRI methodology or brain regions / tissue compartments. These abbreviations are listed here.

MRI acquisition

DWI = Diffusion Weighted Imaging; EPI = Echo-Planar Imaging; FA = Flip Angle; FFE = Fast Field Echo; FLAIR = Fluid-Attenuated Inversion Recovery; FOV = Field of View; FSE = Fast Spin Echo; FSPGR = Fast Spoiled Gradient Echo; GRE = Gradient Echo; HARDI = High Angular Resolution Diffusion Imaging; IR = Inversion Recovery; MPRAGE = Magnetization Prepared – Rapid Gradient Echo; NEX = Number of Excitations; PD = Proton Density; SPGR = Spoiled Gradient Echo; TE = Echo Time; TI = Inversion Time; TR = Repetition Time.

MRI analysis

AFNI = Analysis of Functional NeuroImages; ANIMAL = Automated Nonlinear Image Matching and Anatomical Labelling; ART = Automated Registration Toolbox; DARTEL = Diffeomorphic Anatomical Registration Through Exponentiated Lie Algebra; DK Atlas = Desikan-Killany Atlas; DPARSF = Data Processing Assistant for Resting-State fMRI; DVARS = spatial standard deviation of successive difference images (D for temporal derivative, VARS for root mean square variance over voxels); DTI = Diffusion Tensor Imaging; FACT = Fiber Assignment by Continuous Tracking; FD = Framewise Displacement; FSL = FMRIB Software Library; MAGeT = Multiple Automatically Generated Templates; MarsBaR = MARSeille Boîte À Région d'Intérêt; MCFLIRT = Motion Correction using FMRIB's Linear Image Registration Tool; MNI = Montreal Neurological Institute; N3 = Non-Parametric Non-Uniform Intensity Normalization; NBS = Network Based Statistic; PAGANI Toolkit = Parallel Graph-Theoretical Analysis Toolkit; PPI = Psychophysiological Interaction; REST = Resting State fMRI Data Analysis Toolkit; ROI = Region of Interest; SPM = Statistical Parametric Mapping; TBSS = Tract Based Spatial Statistics; VBM = Voxel-based morphometry; 3dLME = AFNI Group Analysis Program with Linear Mixed-Effects Modeling.

Brain regions and brain tissue compartments

ACC = Anterior Cingulate Cortex; CC = Corpus Callosum; CT = Cortical Thickness; CSF = Cerebrospinal Fluid; DMN = Default Mode Network; DLPFC = Dorsolateral Prefrontal Cortex; FG = Fusiform Gyrus; FP = Frontal Pole; GM = Gray Matter; ICG = Inferior Occipital Gyrus; IFG = Inferior Frontal Gyrus; IOG = Inferior Occipital Gyrus; IPL = Inferior Parietal Lobule; ITG = Inferior Temporal Gyrus; LG = Lingual Gyrus; MFG = Middle Frontal Gyrus; MPFC = Medial Prefrontal Cortex; MTG = Middle Temporal Gyrus; NA = Nucleus Accumbens; OFC = Orbitofrontal Cortex; PFC = Prefrontal Cortex; PCC = Posterior Cingulate Cortex; SA = Surface Area; SFG = Superior Frontal Gyrus; SMA = Supplementary Motor Area; SMG = Supra-Marginal Gyrus; SPG = Superior Parietal Gyrus; SPL = Superior Parietal Lobule; STG = Superior Temporal Gyrus; STS = Superior Temporal Sulcus; TP = Temporal Pole; TPJ = Temporoparietal Junction; HG = Heschl's Gyrus (Transverse Temporal Gyrus); VLPFC = Ventrolateral Prefrontal Cortex.

References

Anticevic, A., Tang, Y., Cho, Y.T., Repovs, G., Cole, M.W., Savic, A., Wang, F., Krystal, J.H., Xu, K., 2014. Amygdala connectivity differs among chronic, early course, and individuals at risk for developing schizophrenia. Schizophr. Bull. 40, 1105–16.

Chang, M., Womer, F.Y., Bai, C., Zhou, Q., Wei, S., Jiang, X., Geng, H., Zhou, Y., Tang, Y., Wang, F., 2016. Voxel-based morphometry in individuals at genetic high risk for schizophrenia and patients with schizophrenia during their first episode of psychosis. PLoS One 11.

Choi, J.S., Park, J.Y., Jung, M.H., Jang, J.H., Kang, D.H., Jung, W.H., Han, J.Y., Choi, C.H., Hong, K.S., Kwon, J.S., 2012. Phase-specific brain change of spatial working memory processing in genetic and ultra-high risk groups of schizophrenia. Schizophr Bull 38, 1189–1199.

Ćurčić-Blake, B., Ford, J.M., Hubl, D., Orlov, N.D., Sommer, I.E., Waters, F., Allen, P., Jardri, R., Woodruff, P.W., David, O., Mulert, C., Woodward, T.S., Aleman, A., 2017. Interaction of language, auditory and memory brain networks in auditory verbal hallucinations. Prog. Neurobiol. 148, 1–20.

de Wit, S., Wierenga, L.M., Oranje, B., Ziermans, T.B., Schothorst, P.F., Engeland, H. Van, Kahn, R.S., Durston, S., 2016. Brain development in adolescents at ultra-high risk for psychosis: Longitudinal changes related to resilience. NeuroImage Clin. 12, 542–549.

DeVylder, J.E., Muchomba, F.M., Gill, K.E., Ben-David, S., Walder, D.J., Malaspina, D., Corcoran, C.M., 2014. Symptom trajectories and psychosis onset in a clinical high-risk cohort: The relevance of subthreshold thought disorder. Schizophr. Res. 159, 278–283.

Frangou, S., 2012. Brain structural and functional correlates of resilience to Bipolar Disorder. Front Hum Neurosci 5, Article 184.

Greenstein, D., Lenroot, R., Clausen, L., Chavez, A., Vaituzis, A.C., Tran, L., Gogtay, N., Rapoport, J., 2011. Cerebellar development in childhood onset schizophrenia and non-psychotic siblings. Psychiatry Res. - Neuroimaging 193, 131–137.

Katagiri, N., Pantelis, C., Nemoto, T., Tsujino, N., Saito, J., Hori, M., Yamaguchi, T., Funatogawa, T., Mizuno, M., 2019. Longitudinal changes in striatum and sub-threshold positive symptoms in individuals with an 'at risk mental state' (ARMS). Psychiatry Res. Neuroimaging 285, 25–30.

Katagiri, N., Pantelis, C., Nemoto, T., Tsujino, N., Saito, J., Hori, M., Yamaguchi, T., Funatogawa, T., Mizuno, M., 2018. Symptom recovery and relationship to structure of corpus callosum in individuals with an 'at risk mental state.' Psychiatry Res. Neuroimaging 272, 1–6.

Katagiri, N., Pantelis, C., Nemoto, T., Zalesky, A., Hori, M., Shimoji, K., Saito, J., Ito, S., Dwyer, D.B., Fukunaga, I., Morita, K., Tsujino, N., Yamaguchi, T., Shiraga, N., Aoki, S., Mizuno, M., 2015. A longitudinal study investigating sub-threshold symptoms and white matter changes in individuals with an 'at risk mental state' (ARMS). Schizophr. Res. 162, 7–13.

Kempton, M.J., Haldane, M., Jogia, J., Grasby, P.M., Collier, D., Frangou, S., 2009. Dissociable Brain Structural Changes Associated with Predisposition, Resilience, and Disease Expression in Bipolar Disorder. J Neurosci 29, 10863–10868.

Kim, S.N., Park, J.S., Jang, J.H., Jung, W.H., Shim, G., Park, H.Y., Hwang, J.Y., Choi, C.-H., Kang, D.-H., Lee, J.-M., Kwon, J.S., 2012. Increased white matter integrity in the corpus callosum in subjects with high genetic loading for schizophrenia. Prog. Neuro-Psychopharmacology Biol. Psychiatry 37, 50–55.

Levy, D.L., Coleman, M.J., Sung, H., Ji, F., Matthysse, S., Mendell, N.R., Titone, D., 2010. The genetic basis of thought disorder and language and communication disturbances in schizophrenia. J. Neurolinguistics 23, 176–192.

Nieto-Castanon, A., 2020. Handbook of functional connectivity Magnetic Resonance Imaging methods in CONN. Hilbert Press.

Strik, W., Dierks, T., Hubl, D., Horn, H., 2008. HaIlucinations, Thought Disorders, and the Language Domain in Schizophrenia. Clin. EEG Neurosci. 39, 91–94.

van Erp, T.G.M., Thompson, P.M., Kieseppä, T., Bearden, C.E., Marino, A.C., Hoftman, G.D., Haukka, J., Partonen, T., Huttunen, M., Kaprio, J., Lönnqvist, J., Poutanen, V.-P., Toga, A.W., Cannon, T.D., 2012. Hippocampal morphology in lithium and non-lithium-treated bipolar I disorder patients, non-bipolar co-twins, and control twins. Hum. Brain Mapp. 33, 501–510.

Vita, A., Dieci, M., Giobbio, G.M., Caputo, A., Ghiringhelli, L., Comazzi, M., Garbarini, M., Mendini, A.P., Morganti, C., Tenconi, F., Cesana, B., Invernizzi, G., 1995. Language and thought disorder in schizophrenia: brain morphological correlates. Schizophr Res 15, 243–

251.
Whitfield-Gabrieli, S., Nieto-Castanon, A., 2012. Conn: A functional connectivity toolbox for correlated and anticorrelated brain networks. Brain 2, 125–141.
Yeo, T.B.T., Krienen, F.M., Sepulcre, J., Sabuncu, M.R., Lashkari, D., Hollinshead, M., Roffman, J.L., Smoller, J.W., Zöllei, L., Polimeni, J.R., Fischl, B., Liu, H., Buckner, R.L., 2011. The organization of the human cerebral cortex estimated by intrinsic functional connectivity. J Neurophysiol 106, 1125–1165.

Table S1. Critical Evaluation

First author	Year	Structural / Functional MRI	Imaging Modality	Region-Specific Results	Critical evaluation and Quality Adjustment Weighting			
					Sample Size Concern	Mult. Comparison Concern	Motion Correction Concern	Quality-Weighting
Fornito	2008	Structural	sMRI	Yes	0	0	0	1
Habets	2008	Structural	sMRI	Yes	0	0	0	1
Kempton	2009	Structural	sMRI	Yes	0	0	0	1
Greenstein	2011	Structural	sMRI	Yes	0	1	0	0.667
Frangou	2012	Structural	sMRI	Yes	0	0	0	1
van Erp	2012	Structural	sMRI	Yes	1	0	0	0.667
Eker	2014	Structural	sMRI	Yes	0	0	0	1
Chakravarty	2015	Structural	sMRI	No	0	0	0	N/A
Goghari	2015	Structural	sMRI	Yes	0	1	0	0.667
Sariçiçek	2015	Structural	sMRI	Yes	0	0	0	1
Zalesky	2015	Structural	sMRI	No	0	0	0	N/A
Chang	2016	Structural	sMRI	Yes	0	0	0	1
de Wit	2016	Structural	sMRI	Yes	1	1	0	0.333
Katagiri	2018	Structural	sMRI	Yes	1	1	0	0.333
Katagiri	2019	Structural	sMRI	Yes	1	1	0	0.333
Yalin	2019	Structural	sMRI	Yes	0	0	0	1
Hoptman	2008	Structural	DWI	Yes	0	0	0	1
Kim	2012	Structural	DWI	Yes	1	1	0	0.333
Boos	2013	Structural	DWI	No	0	1	0	N/A
Goghari	2014	Structural	DWI	No	0	0	0	N/A
Katagiri	2015	Structural	DWI	Yes	1	0	0	0.667
Fusar-Poli	2010	Functional	Task-fMRI (Working Memory)	Yes	1	0	0	0.667
Fusar-Poli	2011	Functional	Task-fMRI (Working Memory)	Yes	1	0	0	0.667
Choi	2012	Functional	Task-fMRI (Working Memory)	Yes	1	0	0	0.667
Smieskova	2012	Functional	Task-fMRI (Working Memory)	Yes	1	0	0	0.667
Stablein	2018	Functional	Task-fMRI (Working Memory)	Yes	0	0	0	1
Pompei	2011	Functional	Task-fMRI (Cognitive Control)	Yes	0	0	1	0.667
Spilka	2015	Functional	Task-fMRI (Emotion Processing)	Yes	0	0	0	1
Sepede	2015	Functional	Task-fMRI (Emotion Processing)	Yes	1	0	0	0.667
Tseng	2015	Functional	Task-fMRI (Emotion Processing)	Yes	1	1	0	0.333
Dima	2016	Functional	Task-fMRI (Emotion Processing)	Yes	0	0	1	0.667
Welge	2016	Functional	Task-fMRI (Emotion Processing)	Yes	0	0	0	1
Spilka	2017	Functional	Task-fMRI (Emotion Processing)	Yes	0	0	0	1
Wiggins	2017	Functional	Task-fMRI (Emotion Processing)	Yes	0	0	0	1
Nimarko	2019	Functional	Task-fMRI (Emotion Processing)	Yes	0	0	0	1
Brüne	2011	Functional	Task-fMRI (Theory of Mind)	Yes	1	0	1	0.333
Willert	2015	Functional	Task-fMRI (Theory of Mind)	Yes	0	0	0	1
Anticevic	2014	Functional	Rs-fMRI	Yes	0	0	0	1
Guo	2014	Functional	Rs-fMRI	No	0	0	0	N/A
Doucet	2017	Functional	Rs-fMRI	Yes	0	0	0	1
Duan	2018	Functional	Rs-fMRI	No	0	0	0	N/A
Ganella	2018	Functional	Rs-fMRI	No	1	0	0	N/A
Guo	2020	Functional	Rs-fMRI	No	0	0	0	N/A

Table S2. Mapped results - Cortical - Left Hemisphere

First author	Year	Banks of the STS	Caudal ACC	Caudal MFG	Cuneus cortex	Entorhinal cortex	FG	Inferior parietal cortex	ITG	Isthmus-cingulate cortex	Lateral occipital cortex	Lateral OFC	LG	Medial OFC	MTG	Parahippocampal gyrus	Paracentral lobule	Pars opercularis	Pars orbitalis	Pars triangularis	Pericalcarine cortex	Postcentral gyrus	PCC	Precentral gyrus	Precuneus cortex	Rostral ACC	Rostral MFG	SFG	SPG	STG	SMG	FP	Temporal pole	HG	Insula
Fornito	2008	0	0	0	0	0	0	0	0	0	0	0	0	0	0	0	0	0	0	0	0	0	0	0	0	1	0	0	0	0	0	0	0	0	0
Habets	2008	0	0	0	0	0	0	0	0	0	0	0	0	0	0	0	0	0	0	0	0	0	0	0	0	0	0	1	0	0	0	0	0	0	0
Kempton	2009	0	0	0	0	0	0	0	0	0	0	0	0	0	0	0	0	0	0	0	0	0	0	0	0	0	0	0	0	0	0	0	0	0	0
Greenstein	2011	0	0	0	0	0	0	0	0	0	0	0	0	0	0	0	0	0	0	0	0	0	0	0	0	0	0	0	0	0	0	0	0	0	0
Frangou	2012	0	0	0	0	0	0	0	0	0	0	0	0	0	0	0	0	0	0	0	0	0	0	0	0	0	0	0	0	0	0	0	0	0	0
van Erp	2012	0	0	0	0	0	0	0	0	0	0	0	0	0	0	0	0	0	0	0	0	0	0	0	0	0	0	0	0	0	0	0	0	0	0
Eker	2014	0	0	0	0	0	0	0	0	0	0	0	0	0	0	0	0	0	0	0	0	0	0	0	0	0	1	0	0	0	0	0	0	0	0
Goghari	2015	0	0	0.7	0.7	0	0.7	0	0	0.7	0	0.7	0.7	0	0	0	0	0.7	0	0.7	0	0	0	0	0.7	0	0	0	0	0.7	0	0	0	0	0
Sariçiçek	2015	0	0	0	0	0	0	0	0	0	0	0	0	0	0	1	0	0	0	0	0	0	0	0	0	0	0	0	0	0	1	0	0	0	0
Chang	2016	0	0	0	0	0	0	0	0	0	0	0	0	0	0	0	0	0	0	0	0	0	0	0	0	0	0	0	0	0	0	0	0	0	0
de Wit	2016	0.3	0	0.3	0	0	0.3	0	0	0	0	0.3	0	0	0.3	0	0	0	0.3	0	0	0	0	0	0.3	0	0.3	0.3	0.3	0	0.3	0	0	0	0
Katagiri	2018	0	0	0	0	0	0	0	0	0	0	0	0	0	0	0	0	0	0	0	0	0	0	0	0	0	0	0	0	0	0	0	0	0	0
Katagiri	2019	0	0	0	0	0	0	0	0	0	0	0	0	0	0	0	0	0	0	0	0	0	0	0	0	0	0	0	0	0	0	0	0	0	0
Yalin	2019	0	0	0	0	0	0	0	0	0	0	0	0	0	0	0	0	0	0	0	0	0	0	0	0	0	0	0	0	0	0	0	0	0	0
Hoptman	2008	0	0	0	0	0	0	0	0	0	0	0	0	0	0	0	0	0	0	0	0	0	0	0	0	1	0	0	0	0	0	0	0	0	0
Kim	2012	0	0	0	0	0	0	0	0	0	0	0	0	0	0	0	0	0	0	0	0	0	0	0	0	0	0	0	0	0	0	0	0	0	0
Katagiri	2015	0	0	0	0	0	0	0	0	0	0	0	0	0	0	0	0	0	0	0	0	0	0	0	0	0	0	0	0	0	0	0	0	0	0
Fusar-Poli	2010	0	0	0	0	0	0	0	0	0	0	0	0	0	0	0	0	0	0	0	0	0	0	0	0	0	0	0	0	0	0	0	0	0	0
Fusar-Poli	2011	0	0	0	0	0	0	0	0	0	0	0	0.7	0	0	0	0	0	0	0	0	0	0	0	0	0	0	0	0.7	0	0	0	0	0	0

Choi	2012	0	0	0	0	0	0	0	0	0	0	0	0	0	0	0	0	0	0	0	0	0	0	0	0	0	0	0	0	0	0	0	0	0	0
Smieskova	2012	0	0	0	0	0	0	0	0	0	0	0	0	0	0	0	0	0	0	0	0	0	0	0	0	0	0	0	0	0	0	0	0	0	0
Stablein	2018	0	0	0	0	0	0	0	0	0	0	0	0	0	0	0	0	0	0	0	0	0	0	0	0.7	0	0	0	0	0	0	0	0	0	0
Pompei	2011	0	0	0	0	0	0	0	0	0	0	0	0	0	0	0	0	0	0	0	0	0	0	0	0	0	0.7	0	0	0	0	0	0	0	0.7
Spilka	2015	0	0	0	0	0	0	0	0	0	0	1	0	1	0	0	0	0	0	1	0	0	0	0	0	0	0	0	0	0	0	0	0	0	0
Sepede	2015	0	0	0	0	0	0	0	0	0	0	0	0	0	0	0	0	0	0	0	0	0	0	0	0	0	0	0	0	0	0	0	0	0	0
Tseng	2015	0	0	0	0	0	0	0	0	0	0	0	0	0	0	0	0	0	0	0	0	0	0	0	0	0	0	0	0	0	0	0	0	0	0
Dima	2016	0	0	0	0	0	0.7	0	0	0	0.7	0	0	0	0	0	0	0	0	0	0	0	0	0	0	0	0	0	0	0	0	0	0	0	0
Welge	2016	0	0	0	0	0	0	0	0	0	0	0	0	0	0	0	0	1	0	0	0	0	0	0	0	0	0	0	0	0	0	0	0	0	0
Spilka	2017	0	0	0	1	0	0	0	0	0	0	0	0	0	0	0	0	0	0	0	0	0	0	0	1	0	0	0	0	0	0	0	0	0	0
Wiggins	2017	0	0	0	0	0	1	1	0	0	0	0	0	0	0	0	0	1	1	1	0	0	1	0	1	0	0	1	0	0	1	0	1	0	1
Nimarko	2019	0	0	0	0	0	1	1	0	0	0	0	1	0	1	0	0	0	1	0	0	0	0	0	1	0	1	0	0	0	0	0	0	0	1
Brüne	2011	0	0	0	0	0	0	0	0	0	0	0	0	0	0.3	0	0	0.3	0.3	0.3	0	0	0	0	0	0	0	0	0	0.3	0.3	0	0	0.3	0
Willert	2015	0	0	0	0	0	0	0	0	0	0	0	0	0	0	0	0	0	0	0	0	0	0	0	0	0	0	0	0	0	0	0	0	0	0
Anticevic	2014	0	0	0	0	0	0	0	0	0	0	0	0	0	0	0	0	0	0	0	0	0	0	0	0	0	0	0	0	0	0	0	0	0	0
Doucet	2017	0	0	0	0	0	0	0	0	0	0	0	0	0	0	0	0	0	1	0	0	0	0	0	0	1	0	0	0	0	0	0	0	0	0
**Total all**		**0.3**	**0**	**1.3**	**2**	**0**	**4**	**2**	**0**	**1**	**0.7**	**2.3**	**2.7**	**1**	**1.7**	**1**	**0**	**3.3**	**3.7**	**3.3**	**0**	**0**	**1**	**0**	**5**	**3**	**3**	**2.3**	**1**	**1.3**	**2.7**	**0**	**1**	**0.3**	**2.7**

Table S2 - continued. Mapped Results - Cortical - Right Hemisphere

**First author**	**Year**	**Banks of the STS**	**Caudal ACC**	**Caudal MFG**	**Cuneus cortex**	**Entorhinal cortex**	**FG**	**Inferior parietal cortex**	**ITG**	**Isthmus-cingulate cortex**	**Lateral occipital cortex**	**Lateral OFC**	**LG**	**Medial OFC**	**MTG**	**Parahippocampal gyrus**	**Paracentral lobule**	**Pars opercularis**	**Pars orbitalis**	**Pars triangularis**	**Pericalcarine cortex**	**Postcentral gyrus**	**PCC**	**Precentral gyrus**	**Precuneus cortex**	**Rostral ACC**	**Rostral MFG**	**SFG**	**SPG**	**STG**	**SMG**	**FP**	**Temporal pole**	**HG**	**Insula**
Fornito	2008	0	0	0	0	0	0	0	0	0	0	0	0	0	0	0	0	0	0	0	0	0	0	0	0	1	0	0	0	0	0	0	0	0	0
Habets	2008	0	0	0	0	0	0	0	0	0	0	0	0	0	0	0	0	0	0	0	0	0	0	0	0	0	0	1	0	0	0	0	0	0	0
Kempton	2009	0	0	0	0	0	0	0	0	0	0	0	0	0	0	0	0	0	0	0	0	0	0	0	0	0	0	0	0	0	0	0	0	0	0

Greenstein	2011	0	0	0	0	0	0	0	0	0	0	0	0	0	0	0	0	0	0	0	0	0	0	0	0	0	0	0	0	0	0	0	0	0	0
Frangou	2012	0	0	0	0	0	0	0	0	0	0	0	0	0	0	0	0	0	0	0	0	0	0	0	0	0	0	0	0	0	0	0	0	0	0
van Erp	2012	0	0	0	0	0	0	0	0	0	0	0	0	0	0	0	0	0	0	0	0	0	0	0	0	0	0	0	0	0	0	0	0	0	0
Eker	2014	0	0	0	0	0	0	0	0	0	0	0	0	0	0	0	0	0	0	0	0	0	0	0	0	0	0	0	0	0	0	0	0	0	0
Goghari	2015	0	0	0.7	0.7	0	0	0	0	0.7	0	0	0.7	0	0	0	0	0.7	0	0.7	0	0	0	0	0.7	0	0	0	0	0.7	0	0	0	0	0
Sariçiçek	2015	0	0	0	0	0	0	0	0	0	0	0	0	0	0	0	0	0	0	0	0	0	0	0	0	0	0	0	0	0	0	0	0	0	0
Chang	2016	0	0	0	0	0	1	0	1	0	0	0	0	0	0	0	0	0	0	0	0	0	0	1	0	0	0	0	0	0	1	0	0	0	0
de Wit	2016	0	0	0.3	0	0	0.3	0	0.3	0	0	0	0	0	0	0.3	0	0	0	0.3	0	0	0	0	0	0	0	0	0	0	0	0	0	0	0
Katagiri	2018	0	0	0	0	0	0	0	0	0	0	0	0	0	0	0	0	0	0	0	0	0	0	0	0	0	0	0	0	0	0	0	0	0	0
Katagiri	2019	0	0	0	0	0	0	0	0	0	0	0	0	0	0	0	0	0	0	0	0	0	0	0	0	0	0	0	0	0	0	0	0	0	0
Yalin	2019	0	0	0	0	0	0	0	0	0	0	0	0	0	0	0	0	0	0	0	0	0	0	0	0	0	0	0	0	1	0	0	0	0	0
Hoptman	2008	0	0	0	0	0	0	0	0	0	0	0	0	0	0	0	0	0	0	0	0	0	0	0	0	0	1	1	0	0	0	0	0	0	0
Kim	2012	0	0	0	0	0	0	0	0	0	0	0	0	0	0	0	0	0	0	0	0	0	0	0	0	0	0	0	0	0	0	0	0	0	0
Katagiri	2015	0	0	0	0	0	0	0	0	0	0	0	0	0	0	0	0	0	0	0	0	0	0	0	0	0	0	0	0	0	0	0	0	0	0
Fusar-Poli	2010	0	0	0	0	0	0	0	0	0	0	0	0	0	0	0	0	0	0	0	0	0	0	1	0	0	0	0	0	0	0	0	0	0	1
Fusar-Poli	2011	0	0	0	0	0	0	0	0	0	0	0	0	0	0	0	0	0	0	0	0	0	0	0	0	0	0	0	0	0	0	0	0	0	0
Choi	2012	0	0	0	0	0	0	0	0	0	0	0	0	0	0	0.7	0	0	0	0	0	0	0	0	0	0.7	0	0	0	0	0	0	0	0	0
Smieskova	2012	0	0	0	0	0	0	0	0	0	0	0	0	0	0	0	0	0.7	0	0	0	0	0	0	0	0	0.7	0	0	0	0	0	0	0	0
Stablein	2018	0	0	0	0	0	0	0	0	0	0	0	0	0	0	0	0	0	0.7	0	0	0	0	0	0.7	0	0	0	0	0	0	0	0	0	0.7
Pompei	2011	0	0	0	0	0	0	0	0	0	0	0	0	0	0	0	0	0	0.7	0	0	0	0	0	0	0	0.7	0	0	0	0	0	0	0	0.7
Spilka	2015	0	0	0	0	0	0	0	0	0	0	0	0	0	0	0	0	0	0	0	0	0	0	0	0	0	0	0	0	0	0	0	0	0	0
Sepede	2015	0	0	0	0	0	0	0	0	0	0	0	0.7	0	0	0	0	0	0	0	0	0	0	0	0	0	0	0.7	0	0	0	0	0	0	0
Tseng	2015	0	0	0	0	0	0	0	0	0	0	0	0	0	0	0.3	0	0	0	0	0	0	0	0	0	0	0	0	0	0	0	0	0	0	0
Dima	2016	0	0	0	0	0	0.7	0	0	0	0.7	0	0	0	0	0	0	0	0	0	0	0	0	0	0	0	0	0	0	0	0	0	0	0	0

Welge	2016	0	0	0	0	0	0	0	0	0	0	0	0	0	0	0	0	0	0	0	0	0	0	0	0	0	0	0	0	0	0	1	0	0	0
Spilka	2017	0	0	0	0	0	0	0	0	0	0	0	0	0	0	0	0	0	0	0	0	0	1	0	1	0	0	0	0	0	0	0	0	0	0
Wiggins	2017	0	0	0	0	0	0	1	0	0	0	0	0	0	0	0	0	1	1	1	0	0	1	0	1	0	0	1	0	0	0	0	1	0	1
Nimarko	2019	0	0	0	0	0	0	1	0	0	0	0	0	0	0	0	0	0	0	0	0	0	0	0	1	0	0	0	0	0	1	0	0	0	0
Brüne	2011	0	0	0	0	0	0	0	0	0	0	0	0	0	0	0	0	0	0	0	0	0	0	0	0	0	0	0	0	0.3	0.3	0	0	0	0
Willert	2015	0	0	0	0	0	0	0	0	0	0	0	0	0	1	0	0	0	0	0	0	0	0	0	0	0	0	1	0	0	0	0	0	0	0
Anticevic	2014	0	0	0	0	0	0	0	0	0	0	0	0	0	0	0	0	0	0	0	0	0	0	0	0	0	0	0	0	0	0	0	0	0	0
Doucet	2017	0	0	0	0	0	1	1	0	0	0	0	0	0	0	0	0	0	0	0	0	0	0	0	0	1	0	0	0	0	0	0	0	0	0
**Total all**		**0**	**0**	**1.3**	**1**	**0**	**3**	**3**	**1.3**	**1**	**0.7**	**0**	**1.7**	**0**	**1**	**1.3**	**0**	**2.7**	**2.3**	**2.3**	**0**	**0**	**2**	**2**	**4.7**	**2.7**	**2.3**	**4.7**	**0**	**2.3**	**2.3**	**1**	**1**	**0**	**3.3**

Table S3. Mapped Results - Subcortical – Bilateral

**First author**	**Year**	**Left Thalamus**	**Left Caudate**	**Left Putamen**	**Left Pallidum**	**Left Hippocampus**	**Left Amygdala**	**Left Accumbens**	**Right Thalamus**	**Right Caudate**	**Right Putamen**	**Right Pallidum**	**Right Hippocampus**	**Right Amygdala**	**Right Accumbens**	**Cerebellum**	**Corpus Callosum**
Fornito	2008	0	0	0	0	0	0	0	0	0	0	0	0	0	0	0	0
Habets	2008	0	0	0	0	0	0	0	0	0	0	0	0	0	0	0	0
Kempton	2009	0	0	0	0	0	0	0	0	0	0	0	0	0	0	1	0
Greenstein	2011	0	0	0	0	0	0	0	0	0	0	0	0	0	0	0.7	0
Frangou	2012	0	0	0	0	0	0	0	0	0	0	0	0	0	0	1	0
van Erp	2012	0	0	0	0	0	0	0	0	0	0	0	0.7	0	0	0	0
Eker	2014	0	0	0	0	0	0	0	0	0	0	0	0	0	0	0	0
Goghari	2015	0	0	0	0	0	0	0	0	0	0	0	0	0	0	0	0
Sariçiçek	2015	0	0	0	0	0	0	0	0	0	0	0	0	0	0	0	0
Chang	2016	0	0	0	0	0	0	0	0	0	0	0	0	0	0	1	0
de Wit	2016	0	0	0	0.3	0	0	0	0	0	0	0	0	0	0	0	0
Katagiri	2018	0	0	0	0	0	0	0	0	0	0	0	0	0	0	0	0.3
Katagiri	2019	0	0	0.3	0	0	0	0	0	0	0.3	0	0	0	0.3	0	0
Yalin	2019	0	0	0	0	0	0	0	0	0	0	0	0	0	0	0	0
Hoptman	2008	0	0	0	0	0	0	0	0	0	0	0	0	0	0	0	0
Kim	2012	0	0	0	0	0	0	0	0	0	0	0	0	0	0	0	0.3
Katagiri	2015	0	0	0	0	0	0	0	0	0	0	0	0	0	0	0	0.7
Fusar-Poli	2010	0	0	0	0	0	0	0	0	0	0	0	0	0	0	0	0
Fusar-Poli	2011	0	0	0	0	0	0	0	0	0	0	0	0	0	0	0	0
Choi	2012	0.7	0	0	0	0	0	0	0	0	0	0	0	0	0	0	0

Smieskova	2012	0	0	0	0	0	0	0	0	0	0	0	0	0	0	0	0
Stablein	2018	0	0	0	0	0	0	0	0	0	0	0	0	0	0	0	0
Pompei	2011	0	0	0	0	0	0	0	0	0	0	0	0	0	0	0	0
Spilka	2015	0	0	0	0	0	0	0	0	0	0	0	0	0	0	0	0
Sepede	2015	0	0	0	0	0	0	0	0	0	0	0	0	0	0	0	0
Tseng	2015	0	0	0	0	0	0	0	0	0	0	0	0	0	0	0	0
Dima	2016	0	0	0	0	0	0	0	0	0	0	0	0	0	0	0	0
Welge	2016	0	0	0	0	0	0	0	0	0	0	0	0	0	0	0	0
Spilka	2017	0	0	0	0	0	0	0	0	0	0	0	0	0	0	0	0
Wiggins	2017	0	0	0	0	0	0	0	0	0	0	0	0	0	0	0	0
Nimarko	2019	0	0	0	0	0	0	0	0	0	0	0	0	0	0	0	0
Brüne	2011	0	0	0	0	0	0	0	0	0	0	0	0	0	0	0	0
Willert	2015	0	0	0	0	0	0	0	0	0	0	0	0	0	0	0	0
Anticevic	2014	0	0	0	0	0	1	0	0	0	0	0	0	1	0	0	0
Doucet	2017	0	0	0	0	0	0	0	0	0	0	0	0	0	0	0	0
**Total all**		**0.7**	**0**	**0.3**	**0.3**	**0**	**1**	**0**	**0**	**0**	**0.3**	**0**	**0.7**	**1**	**0.3**	**3.7**	**1.7**

Table S4. DK-atlas mapping details

**First author**	**Year**	**Region showing resilience-effect as reported in paper**	**Corresponding DK-atlas region**	**Remarks**
Fornito	2008	L/R Rostral Limbic ACC	L/R Rostral ACC	
Habets	2008	L/R SFG	L/R SFG	
Kempton	2009	L Cerebellar Vermis	N/A (Cerebellum)	
Greenstein	2011	L/R Superior Cerebellar Vermis	N/A (Cerebellum)	
Frangou	2012	L/R Cerebellar Vermis	N/A (Cerebellum)	
van Erp	2012	R Hippocampus	N/A (R Hippocampus)	
Eker	2014	L DLPFC	L Rostral MFG	
Chakravarty	2015	Striatal shape abnormalities	N/A (Striatum)	
Goghari	2015	L/R Caudal MFG	L/R Caudal MFG	
		L/R IFG (Opercular)	L/R Pars Opercularis	
		L/R IFG (Triangular)	L/R Pars Triangularis	
		L/R STG	L/R STG	
		L/R Isthmus-Cingulate Gyrus	L/R Isthmus-Cingulate Cortex	
		L/R Precuneus	L/R Precuneus Cortex	
		L/R Cuneus	L/R Cuneus Cortex	
		L/R LG	L/R LG	
		L FG	L FG	
		L Lateral OFC	L Lateral OFC	
Sariçiçek	2015	L SMG	L SMG	
		L Parahippocampal Gyrus	L Parahippocampal Gyrus	
Zalesky	2015	Occipitotemporal CT correlations	N/A	
Chang	2016	R FG		Discrepancy between text and figure on regions showing higher volume in GHR. We used Fig. 2, which shows higher volume in
		R ITG		
		R SMG		

		R Precental Gyrus		clusters A and H, corresponding to R ITG, FG, and cerebellum (A), and R SMG and precentral gyrus (H).
		R Cerebellum	N/A (Cerebellum)	
de Wit	2016	L/R Caudal MFG	L/R Caudal MFG	
		L/R FG	L/R FG	
		L SFG	L SFG	
		L Rostral MFG	L Rostral MFG	
		L IFG (Orbital)	L Pars Orbitalis	
		L Lateral OFC	L Lateral OFC	
		L MTG	L MTG	
		L Banks of the STS	L Banks of the STS	
		L SMG	L SMG	
		L SPG	L Superior Parietal Cortex	
		L Precuneus	L Precuneus	
		R ITG	R ITG	
		R Parahippocampal Gyrus	R Parahippocampal Gyrus	
		L Pallidum	N/A (L Pallidum)	
Katagiri	2018	Central CC	N/A (Corpus Callosum)	
Katagiri	2019	L/R Putamen	N/A (L/R Putamen)	
		R Nucleus Accumbens	N/A (R Nucleus Accumbens)	
Yalin	2019	R STG	R STG	
Hoptman	2008	L Subgenual ACC	L Rostral ACC	Talairach coordinates of L subgenual ACC reported in paper correspond to L rostral ACC.
		R MFG	R Rostral MFG	
		R SFG	R SFG	
		Pontine Tegmental White Matter	N/A	
Kim	2012	Genu of the CC	N/A (Corpus Callosum)	
Boos	2013	L/R Arcuate Fascicles		
Goghari	2014	R Fimbria of the Fornix		
Katagiri	2015	L Genu of the CC	N/A (Corpus Callosum)	
Fusar-Poli	2010	L LG	L LG	
		L SPL	L Superior Parietal Cortex	
Fusar-Poli	2011	R ACC	R Rostral ACC	Right ACC reported, MNI coordinates suggest closer to rostral than caudal ACC.
Choi	2012	R DLPFC	R Rostral MFG	
		R VLPFC	R Pars Opercularis	R VLPFC was identified as BA 44, corresponding to IFG, pars opercularis.
		L Thalamus	N/A (L Thalamus)	
Smieskova	2012	L/R Precuneus	L/R Precuneus Cortex	
		R IFG	R Pars Orbitalis	R IFG was identified as BA 47 according to MNI coordinates, corresponding to IFG, pars orbitalis.
		R Insula	R Insula	
Stablein	2018	R Insula	R Insula	
		R Precental Gyrus	R Precental Gyrus	

Pompei	2011	L/R DLPFC	L/R Rostral MFG	
		L/R Insula	L/R Insula	
		R VLPFC	R Pars Orbitalis	R VLPFC was identified as BA 47 according to MNI coordinates, corresponding to IFG, pars orbitalis.
Spilka	2015	L IFG	L Pars Triangularis	L IFG was identified as BA 45 according to MNI coordinates, corresponding to IFG, pars triangularis.
		L OFC	L Lateral and Medial OFC	
Sepede	2015	R LG	R LG	
		R SFG	R SFG	
		R Pre-SMA	R SFG	R pre-SMA was identified as BA 6/8 according to MNI coordinates, corresponding to R SFG.
Tseng	2015	R Parahippocampal Gyrus	R Parahippocampal Gyrus	
Dima	2016	L/R FG	L/R FG	
		L/R IOG	L/R Lateral Occipital Cortex	
Welge	2016	L IFG (Opercular)	L Pars Opercularis	
		R Frontal Pole	R Frontal Pole	
Spilka	2017	L/R Precuneus	L/R Precuneus Cortex	
		L Cuneus	L Cuneus Cortex	
		R PCC	R PCC	
Wiggins	2017	L/R IFG	L/R Pars Opercularis	L/R IFG was identified as BA 46
		L/R SFG	L/R SFG	
		L/R PCC	L/R PCC	
		L/R Precuneus	L/R Precuneus Cortex	
		L/R Temporo-Parietal Areas	L/R Inferior Parietal Cortex	L/R temporo-parietal areas were localized to BA 39 (angular gyrus), which is part of inferior parietal cortex in DK-atlas.
		L/R Temporal Pole	L/R Temporal Pole	
		L/R Insula	L/R Insula	
		L FG	L FG	
		L SMG	L SMG	
Nimarko	2019	L/R Precuneus	L/R Precuneus Cortex	
		L IFG	L Pars Orbitalis	L IFG was identified as BA 47 according to MNI coordinates, corresponding to IFG, pars orbitalis.
		L/R Inferior Parietal Lobule	L/R Inferior Parietal Cortex	
		L FG	L FG	
		L LG	L LG	
		R SMG	R SMG	
		L Insula	L Insula	
		L MTG	L MTG	
		L MFG	L Rostral MFG	
Brüne	2011	L IFG	L Pars Triangularis	
		L/R STG	L/R STG	
		L/R Angular Gyrus	L/R SMG	
		L MTG	L MTG	

		L Transverse Temporal Gyrus	L HG	
Willert	2015	R MTG	R MTG	
		R sMFG	R SFG	sMFG refers to superior medial frontal gyrus, corresponding to SFG (which has a large medial section).
Anticevic	2014	L/R Amygdala	N/A (L/R Amygdala)	
		Brainstem	N/A	
Guo	2014	Long- and Short-Range Connectivity	N/A	
Doucet	2017	R FG	R FG	
		L IFG (Orbital)	L Pars Orbitalis	
		R Angular Gyrus	R Inferior Parietal Cortex	
		L/R Ventral ACC	L/R Rostral ACC	Figure in paper suggests rostral ACC
Duan	2018	Reduced distance strength	N/A	
Ganella	2018	47 functional connections	N/A	
Guo	2020	Higher global FC diversity, degree, clustering, efficiency	N/A	

Table S5. Exclusion after full-text assessment – studies and rationale

First author	Year	Reason for exclusion after full-text asssessment
de Wit	2017	No brain markers of resilience per current definition reported
Cannon	2012	No brain markers of resilience per current definition reported
Frangou	2017	No brain markers of resilience per current definition reported
Habel	2004	No brain markers of resilience per current definition reported
Hajek	2013	No brain markers of resilience per current definition reported
Karch	2009	No brain markers of resilience per current definition reported
Koutsouleris	2012	No brain markers of resilience per current definition reported
Unschuld	2014	No brain markers of resilience per current definition reported
van der Leeuw	2017	No brain markers of resilience per current definition reported
Emsell	2014	No brain markers of resilience per current definition reported
Sapara	2014	Sample did not meet inclusion criteria: SCZ preserved vs impaired insight, and HC
Sepede	2010	Sample did not meet inclusion criteria: only FDR and HC
Singh	2014	Sample did not meet inclusion criteria: only FDR and HC
Tan	2006	Sample did not meet inclusion criteria: SCZ, incl high- and low-performers, and HC
Van Leeuwen	2019	Sample did not meet inclusion criteria: only FDR and HC
Weickert	2009	Sample did not meet inclusion criteria: SCZ, incl subset of good learners, and HC
Ladouceur	2013	Sample did not meet inclusion criteria: only FDR BD and HC)
Dazzan	2018	Review / commentary
Lee	2014	Review / meta-analysis
Moran	2013	Review
Ordonez	2016	Review
Ozerdem	2016	Review
Ruhrman	2014	Review
Smieskova	2012	Review
Cattarinussi	2018	Review / meta-analysis
Piguet	2015	Review
